# PTBP1 knockdown impairs autophagy flux and inhibits gastric cancer progression through TXNIP-mediated oxidative stress

**DOI:** 10.1186/s11658-024-00626-1

**Published:** 2024-08-17

**Authors:** Shimin Wang, Xiaolin Wang, Changhong Qin, Ce Liang, Wei Li, Ai Ran, Qiang Ma, Xiaojuan Pan, Feifei Yang, Junwu Ren, Bo Huang, Yuying Liu, Yuying Zhang, Haiping Li, Hao Ning, Yan Jiang, Bin Xiao

**Affiliations:** 1 College of Pharmacy, Chongqing Medical University, Chongqing, 400016 People’s Republic of China; 2 Department of Pharmacy, Chongqing University Cancer Hospital, Chongqing, 400030 People’s Republic of China; 3 Key Laboratory of Basic Pharmacology of Ministry of Education and Joint International Research Laboratory of Ethnomedicine of Ministry of Education, Zunyi Medical University, Zunyi, 563006 Guizhou People’s Republic of China

**Keywords:** GC, Autophagy, PTBP1, TXNIP, Chloroquine

## Abstract

**Background:**

Gastric cancer (GC) is a prevalent malignant tumor, and the RNA-binding protein polypyrimidine tract-binding protein 1 (PTBP1) has been identified as a crucial factor in various tumor types. Moreover, abnormal autophagy levels have been shown to significantly impact tumorigenesis and progression. Despite this, the precise regulatory mechanism of PTBP1 in autophagy regulation in GC remains poorly understood.

**Methods:**

To assess the expression of PTBP1 in GC, we employed a comprehensive approach utilizing western blot, real-time quantitative polymerase chain reaction (RT–qPCR), and bioinformatics analysis. To further identify the downstream target genes that bind to PTBP1 in GC cells, we utilized RNA immunoprecipitation coupled with sequencing (si-PTBP1 RNA-seq). To evaluate the impact of PTBP1 on gastric carcinogenesis, we conducted CCK-8 assays, colony formation assays, and GC xenograft mouse model assays. Additionally, we utilized a transmission electron microscope, immunofluorescence, flow cytometry, western blot, RT–qPCR, and GC xenograft mouse model experiments to elucidate the specific mechanism underlying PTBP1’s regulation of autophagy in GC.

**Results:**

Our findings indicated that PTBP1 was significantly overexpressed in GC tissues compared with adjacent normal tissues. Silencing PTBP1 resulted in abnormal accumulation of autophagosomes, thereby inhibiting GC cell viability both in vitro and in vivo. Mechanistically, interference with PTBP1 promoted the stability of *thioredoxin-interacting protein (TXNIP)* mRNA, leading to increased *TXNIP*-mediated oxidative stress. Consequently, this impaired lysosomal function, ultimately resulting in blockage of autophagic flux. Furthermore, our results suggested that interference with PTBP1 enhanced the antitumor effects of chloroquine, both in vitro and in vivo.

**Conclusion:**

PTBP1 knockdown impairs GC progression by directly binding to *TXNIP* mRNA and promoting its expression. Based on these results, PTBP1 emerges as a promising therapeutic target for GC.

**Graphical Abstract:**

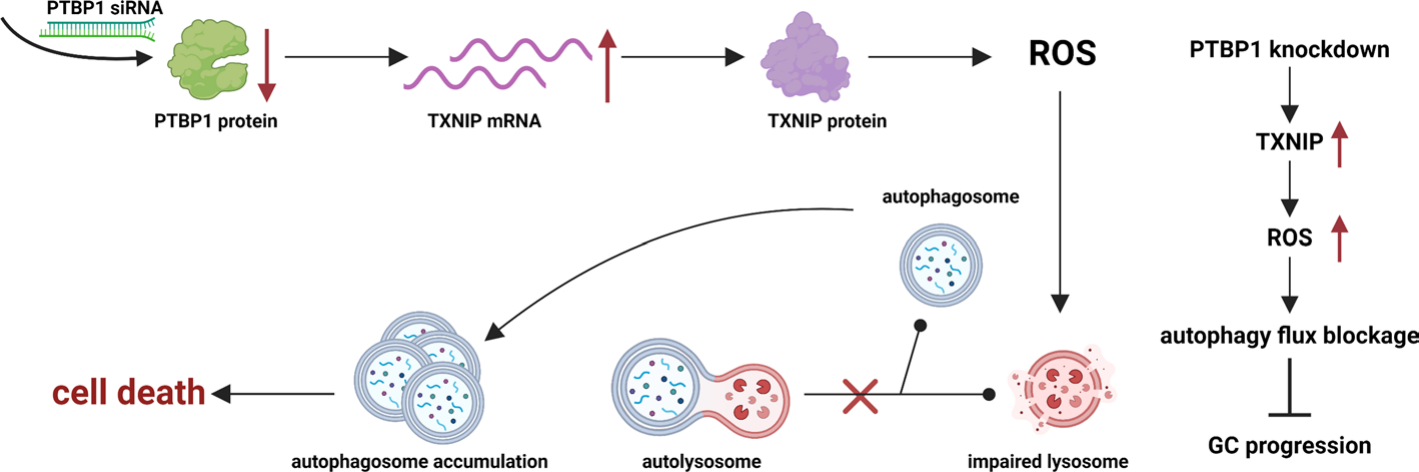

**Supplementary Information:**

The online version contains supplementary material available at 10.1186/s11658-024-00626-1.

## Introduction

Gastric cancer (GC) ranks as the fifth most prevalent cancer worldwide and the third leading cause of cancer-related mortality, accounting for approximately 800,000 deaths annually [1]. The etiology of GC is multifactorial, including *Helicobacter pylori* infection, environmental influences, dietary patterns, genetic predisposition, and precancerous conditions [2]. Although surgical resection remains the primary treatment modality, significant progress has been made in adjuvant therapies such as chemotherapy, targeted therapy and immunotherapy [3]. However, the prognosis for patients with GC remains unfavorable, with 5-year survival rates hovering between 20% and 30% [4]. Therefore, a deeper understanding of the underlying pathogenesis of GC is imperative to identify novel therapeutic targets and ultimately improve patient outcomes.

Aberrant expression of RNA-binding proteins (RBPs) is observed in various human cancers, contributing significantly to tumorigenesis and progression. These proteins have been implicated in diverse biological processes, including apoptosis [5], epithelial-mesenchymal transition (EMT) [6], DNA damage response (DDR) [7], cell proliferation [8, 9], immune response [10], and metabolism [11]. Moreover, they are considered as significant predictors of poor clinical prognosis in cancer patients [12–14]. Polypyrimidine tract-binding protein 1 (PTBP1) is an RNA-binding protein characterized by an N-terminal nuclear shuttle domain and four RNA-recognition motifs (RRMs) [15]. As a key regulator of posttranscriptional gene expression, PTBP1 modulates various aspects of RNA metabolism, including precursor messenger RNA (pre-mRNA) splicing [15, 16] and controls messenger RNA( mRNA) 3′ end processing [17], localization [18], stability [19] and translation [20]. PTBP1 exhibits abnormal expression in various tumors and plays a crucial role in their development [21–23]. It has also been reported that PTBP1 is involved in the proliferation [24], migration [19], and invasion [25] of GC. However, the precise mechanism underlying its function in GC remains poorly understood.

Autophagy, a highly conserved catabolic process, involves the formation of autophagosomes that fuse with lysosomes to degrade misfolded proteins and damaged organelles. Disruptions in this process can lead to the accumulation of autophagosomes and unconsumed damaged organelles, ultimately resulting in cell death. Autophagy plays a dual role in tumorigenesis, with previous reports [26, 27] suggesting that it can inhibit tumor cell proliferation, dissemination, and metastatic potential by inducing oxidative stress and DNA damage, as well as promote tumor cell survival by providing energy and essential compounds under various stress stimuli [28]. However, the precise regulatory mechanisms of autophagy in GC remain unclear, necessitating further in-depth investigation.

In this study, we demonstrated that PTBP1 knockdown significantly inhibited GC cell viability and tumor growth, both in vitro and in vivo. Mechanistically, PTBP1 interacted with *TXNIP* mRNA through its RNA recognition motifs RRM1 and RRM3, which in turn reduced *TXNIP* mRNA stability to inhibit its expression. Consequently, PTBP1 knockdown triggered in *TXNIP*-mediated oxidative stress and led to lysosomal dysfunction and autophagic flux blockage, ultimately causing GC cell death. Furthermore, PTBP1 knockdown enhanced the antitumor effects of the autophagy inhibitor chloroquine in vivo and in vitro. These findings suggest that PTBP1 serves as a regulator of autophagy and may have represented a promising therapeutic target for GC.

## Materials and methods

### Clinical specimens

GC tissues and their corresponding adjacent noncancerous (NC) tissues were obtained from Southwest Hospital of Army Medical University. A total of 20 paired samples were collected (Supporting Information Table S1). None of the included patients received any neoadjuvant therapy prior to surgery, and all were diagnosed with GC based on postoperative pathology. The surgically resected specimens were collected in EP tubes containing RNA Later (Thermo Scientific, Pittsburg, PA, USA) and stored at −80 °C for further analysis. Informed consent was obtained from all the patients, and the study was approved by the Ethical Review Committee of Chongqing Medical University and the Southwest Hospital of Army Medical University.

### Cell lines

The human GC cell lines SGC7901 and HGC-27 were procured from the American Type Culture Collection (ATCC) through the Army Medical University (Chongqing, China). Additionally, AGS cells were purchased from Meisen (CTCC-001-0038, Hangzhou, China). All cells were cultured in either DMEM, F12, or RPMI 1640 medium (basal medium, Shanghai, China) and maintained under standard incubation conditions at 37 °C in a humidified atmosphere with 5% CO_2_.

### Western blot

For total protein extraction, radioimmunoprecipitation assay (RIPA) lysis buffer containing PMSF and phosphatase inhibitors was employed. The protein samples were then resolved using sodium dodecyl sulfate polyacrylamide gel electrophoresis (SDS–PAGE) gels and transferred onto polyvinylidene fluoride membranes (PVDF). Prior to incubation with primary antibodies, the membranes were blocked with 10% skim milk and subsequently incubated overnight at 4 °C with primary antibodies. After thorough washing with PBST, the membranes were incubated with HRP-coupled secondary antibody for 1 h at room temperature. For visualization, ECL chemiluminescence reagent was utilized. The primary antibodies used in this study were diluted in primary antibody dilution buffer at a ratio of 1:1000. These antibodies included β-actin (TA-09, ZSGB-bio, Beijing, China), PTBP1 (#32-4800, Invitrogen, Carlsbad, CA, USA), FLAG (#14793, CST, Danvers, MA, USA), LC3A/B (#12741, CST, Danvers, MA, USA), P62 (#23214, CST, Danvers, MA, USA), and TXNIP (ab188865, Abcam, Cambridge, UK).

### Quantitative real-time PCR (RT–qPCR)

RNA was isolated with RNAiso Plus (Takara, Kyoto, Japan) and subsequently converted into cDNA employing PrimeScript RT premix (Takara, Kyoto, Japan). Real-time quantitative PCR (RT–qPCR) was performed using SYBR Green Master Mix (Bioground, Chongqing, China) on the CFX Connect Real-Time Fluorescence PCR System (BioRad, Hercules, CA, USA). β-actin served as an internal reference to ensure accuracy. The transcript levels were quantified using the Bio-Rad CFX96 Real-Time Fluorescence PCR System and the 2^−ΔΔCT^ method. The qPCR protocol entailed an initial predenaturation step at 95 °C for 30 s, followed by 40 cycles of denaturation at 95 °C for 10 s, annealing at 58 °C (or 61.9 °C for PTBP1) for 10 s, and extension at 72 °C for 10 s. The specific primer sequences are detailed in Table S2.

### Cell viability assay

Cell viability was evaluated using the Cell Counting Kit-8 (CCK-8, Biosharp, Hefei, China). Specifically, 1.5 × 10^3^ cells were plated into 96-well plates with containing 100 μl of complete culture medium and incubated under standard conditions. Subsequently, a mixture of CCK-8 reagent and complete medium in a 1:10 ratio was added to the wells, and the plates were incubated for an additional 2 h under standard conditions. Cell viability was quantified by measuring the absorbance at 450 nm using a Varioskan LUX enzyme labeler (Thermo Scientific, Waltham, MA, USA).

### Cell colony formation assay

A total of 1.5 × 10^3^ cells were inoculated in six-well plates containing 2 ml of complete medium and cultured for 1–2 weeks to allow for the formation of cell spheres. To preserve the cell spheres, they were fixed with a 4% paraformaldehyde solution for 10 min. Following fixation, the spheres were stained with 1% crystal violet for 10 min. The plates were then washed three times with PBS and air-dried at room temperature. Finally, the images of the stained cell spheres were captured using an Epson scanner (Suwa, Nagano, Japan).

### Actinomycin D assay

To assess mRNA stability, GC cells were treated with either small-interfering negative control (si-NC) or si-PTBP and seeded in 12-well plates. Subsequently, the cells were then exposed to actinomycin D (4 μg/ml for AGS and 6 μg/ml for HGC-27) or DMSO for specific periods of time. At the specified timepoints, the cells were lysed using RNAiso Plus (Takara, Kyoto, Japan) to extract RNA for further RT–qPCR analysis.

### Measurement of reactive oxygen species (ROS) levels

Cells were treated with either si-NC or si-PTBP1 for 48 h. Subsequently, the oxidative reaction indicator dichlorodihydrofluorescein diacetate (DCFH-DA, Beyotime, S0033) was applied at a dilution ratio of 1:1000 and incubated with the cells at 37 °C for 30 min. The changes in intracellular ROS levels were then assessed using an inverted fluorescence microscope (Olympus, Tokyo, Japan).

### Transmission electron microscopy

Transmission electron microscopy (TEM) was utilized to visualize autophagosomes. For cell samples, PTBP1-knockdown cells were treated for 48 h and then centrifuged in 1.5 ml pointed bottom EP tubes at 1200 rpm for 10 min. The supernatant was discarded, and a fixative was gently introduced along the tube wall to avoid disruption of the sample clumps. The tubes were stored at 4 °C in a refrigerator. For tissue samples, animals were anesthetized and euthanized, and their subcutaneous tumor tissues, approximately 1 mm^3^ in size, were promptly immersed in the electron microscope fixative. Images were captured using a transmission electron microscope (JEM-1400 plus, Tokyo, Japan).

### Plasmid transfection and detection of GFP-LC3 puncta

The GFP-LC3 plasmid, graciously provided by Dr. Ma from Chuanbei Medical College in Nanchong, China, was transfected into AGS and HGC-27 cells using neofect (Beijing, China), adhering strictly to the manufacturer's protocol. Following 24 h of transfection, the cells were treated with si-NC or si-PTBP1 for 48 h. Subsequently, the transition in the state of green fluorescence was observed under a confocal microscope (Leica Microsystems, Wetzlar, Germany). This transition, from a diffuse state to a punctate state, served as an indication of the formation of autophagic vesicles. The structural map of the GFP-LC3 plasmid is detailed in Figure S1A.

### Immunofluorescence staining

GC cells were treated with either si-NC or si-PTBP and subsequently fixed with 4% paraformaldehyde for 15 min. Following this, the cells were incubated with immunology-staining blocking buffer (consisting of 50 ml PBS, 0.5 g BSA, and 150 ul Triton-100) for 60 min at 37 °C. Next, the cells were incubated with primary antibodies overnight at 4 °C and then stained with secondary antibody (#26567 and #L3016, Signalway Antibody, College Park, MD, USA) for 1 h at room temperature. Afterward, the nuclei were stained with Hoechst 33342 (1 μg/ml) for 10 min. Images were captured with a confocal microscope (Leica Microsystems, Wetzlar, Germany). The primary antibodies used had a dilution ratio of 1:100 and were LC3B (ab192890, Abcam, Cambridge, UK) and LAMP1 (#15665, CST, Danvers, MA, USA).

### Lentivirus infection and quantification of mRFP-GFP-LC3 puncta

Initially, the cells were cultured in 48-well plates and then transfected with mRFP-GFP-LC3 lentivirus (HanBio Technology, Shanghai, China) to establish a constant transfection strain. Posttransfection, the cells were subjected to various treatments: PTBP1 knockdown for 48 h, rapamycin exposure at a concentration of 250 nM, or chloroquine treatment at at a concentration of 20 uM. Nuclear staining was performed using Hoechst 33342 (1 μg/ml), and visualization was achieved using a confocal microscope (Leica Microsystems, Wetzlar, Germany). LC3 was tracked via the mRFP tag, and reduced GFP expression signified the fusion of the autophagosome with the lysosome, culminating in the formation of an autolysosome. This pH change caused the quenching of GFP fluorescence, leaving only red fluorescence detectable. The red and green fluorescence images were merged during microimaging, with autophagosomes represented by yellow dots and autolysosomes represented by red dots. To quantify the intensity of autophagic flux, the number of both yellow and red dots was counted. The structural map of the mRFP-GFP-LC3 lentivirus is detailed in Figure S1B.

### Lyso-tracker red staining

Cells were treated with either si-NC or si-PTBP1 for 24 h, followed by an additional 24 h incubation on iBiDi culture plates (Martin Reid, Germany). Subsequently, 100 μl of Lyso-Tracker Red (75 nM, Beyotime, Chongqing, China) was added to the cells, and they were incubated for 30 min at 37 °C. The cell nuclei were then stained with Hoechst 33342 (1 μg/ml) for 10 min at room temperature. The functional strength of the lysosomes was evaluated by counting the number of red dots observed under a confocal microscope (Leica Microsystems, Wetzlar, Germany).

### Animal experiments

For our in vivo studies, we employed 4-week-old male BALB/c nude mice (GemPharmatech, Chengdu, China). Xenograft tumors were established by injecting 2 × 10^6^ HGC-27 cells suspended in 100 μl serum-free medium into the backs of the mice. The tumor size was measured daily with calipers after 1 week, and tumor volume was calculated according to the formula: length × width^2^/2. Once the xenograft tumors reached the desired volume, the mice were randomly divided into three groups (*n* = 5) for the in vivo salvage assay: control group, PTBP1 knockdown group, and the PTBP1 and TXNIP co-knockdown group. Cholesterol-modified si-NC, si-PTBP1, or si-PTBP1 + si-TXNIP (TSINGKE, 5 nmol/kg) was dissolved in 25 μl of sterile saline and mixed with 5 μl of in vivo transfection reagent (Engreen, Beijing, China). This mixture was then injected into the tumor every 2 days for 2–3 weeks. In experiments involving combined chloroquine treatment, the mice were randomly divided into four groups (*n* = 5): control group, chloroquine-treated group alone, PTBP1-knockdown group, and the PTBP1-knockdown and chloroquine co-treated group. Chloroquine was administered at a concentration of 40 mg/ml in 25 μl, while the siRNA levels remained consistent with those previously mentioned. Additionally, lentivirus containing interfering PTBP1 was also injected into the tumor mass once weekly for a duration of 2 weeks. The animals were euthanized once the tumor size of the nude mice reached an appropriate size. All animal studies were approved by the Ethical Review Committee of Chongqing Medical University.

### Immunohistochemistry assay

Nude mice were euthanized, and their subcutaneous tumor tissues were promptly fixed in 4% paraformaldehyde solution. The fixed tissue blocks were sent to Bios Biotech in Wuhan, China for embedding, sectioning, histochemistry, and white light scanning. For the immunohistochemistry experiments, the following antibodies were utilized: PTBP1 (#32-4800, Invitrogen, Carlsbad, CA, USA), P62 (EM0704, HuaBio, Hangzhou, China), LC3B (ab192890, Abcam, Cambridge, UK), and TXNIP (ab188865, Abcam, Cambridge, UK).

### RIPA

To perform the RIPAs, we employed the Magna RIP kit (Millipore, MA, USA). The cells were processed at a density of 1 × 10^7^ cells per reaction and lysed for 5 min using lysis buffer containing protease and RNase inhibitors. Magnetic beads were conjugated with either 8 μg of PTBP1 antibody (#32–4800, Invitrogen, Carlsbad, CA, USA), Flag (#14793, CST, Danvers, MA, USA), or control IgG at room temperature and then added to the respective cell lysates. The samples were incubated overnight at 4 °C. Following incubation, RNA purification and protein extraction procedures were conducted. The effectiveness of the RIPA technique was confirmed through western blot analysis, while the enrichment of mRNAs was assessed using RT-qPCR.

### RNA sequencing

To elucidate the downstream targets of PTBP1, RNA sequencing was conducted on AGS cells following PTBP1 suppression (Supporting Information Table S3). Furthermore, RNA immunoprecipitation (RIP) and RNA sequencing were performed on SGC-7901 cells to identify RNA molecules that physically interact with the PTBP1 protein, as previously described (Supporting Information Table S4) [19].

### Statistical analysis

Statistical analysis was performed using GraphPad Prism 8 software (GraphPad, San Diego, CA, USA). The Student's *t*-test was used to determine differences between two groups, while Pearson correlation analysis was employed to evaluate the correlation between these groups. A statistical significance value of *P* < 0.05 was considered significant. PTBP1 expression levels in cancer were retrieved from the Gene Expression Profiling Interaction Analysis (GEPIA) (http://gepia2.cancer-pku.cn/#inde; accessed on 28 April 2022). Survival curves of PTBP1 in patients with GC were obtained from Kaplan–Meier Plotter (https://kmplot.com/analysis/; accessed on 16 May 2022). Our investigation included four autophagy-related genes *(ATG10, ATG13, ATG14, LC3*) as features in the GEPIA analysis, and the correlation between these genes and PTBP1 was calculated.

## Results

### PTBP1 knockdown inhibits GC cell viability and may be associated with GC autophagy regulation

**Fig. 1:**
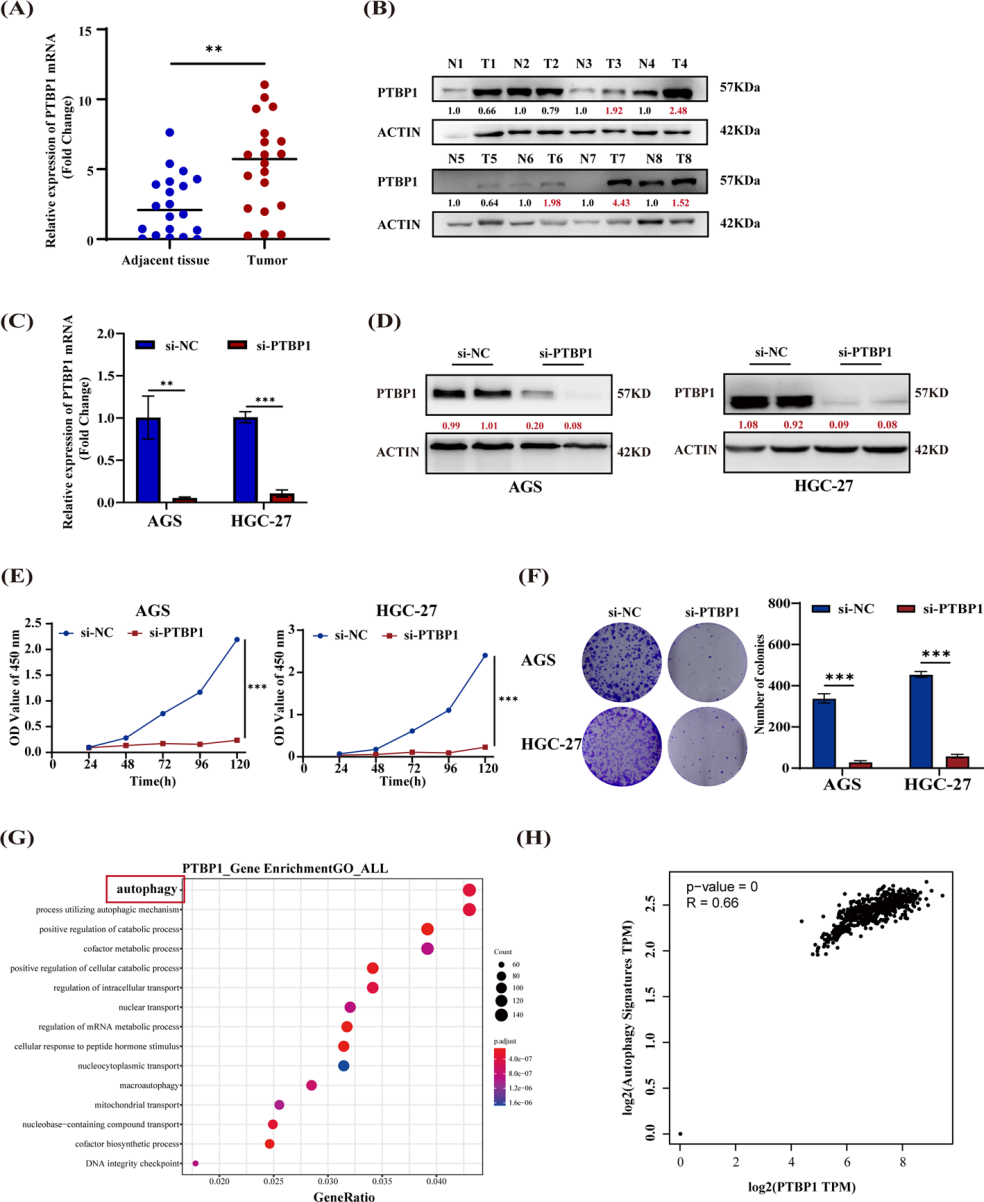
Silencing of PTBP1 inhibits GC cell viability and may be linked to autophagy. **A**,**B** The mRNA (**A**, *n* = 20) expression and protein levels (**B**, *n* = 8) of PTBP1 in GC tissues were measured via RT–qPCR and western blot. β-actin served as a control and densitometric values of band intensity were indicated by numbers. Unpaired student’s *t*‐test, *n* > 3. **P < 0.01. **C**,**D** PTBP1 mRNA (**C**) expression and protein (**D**) levels in AGS and HGC27 cells treated with PTBP1 knockdown by siRNAs. Red numbers represent densitometric values. Unpaired student’s *t*‐test, *n* = 3. **P < 0.01, ***P < 0.001. **E**,**F** CCK-8 (**E**) and plate colony formation (**F**) assays in AGS and HGC-27 cells after stable PTBP1 silencing. Data are presented as mean ± standard deviation (SD). Unpaired student’s *t*‐test, *n* = 3. ***P < 0.001. **G** The Gene Ontology (GO) terms associated with mRNAs bound to PTBP1 protein. **H** Correlation between autophagy-related genes and PTBP1 generated from GEPIA using Pearson correlation. Graphs were obtained from GEPIA

In our previous study, we identified that PTBP1 was one of the differentially expressed RBPs and promoted metastasis in GC [19]. To further investigate the role of PTBP1 in GC, we initially verified the upregulation of *PTBP1* mRNA and protein levels in more GC tumor tissues through RT–qPCR and western blot analysis (Fig. 1A, B). Subsequently, we designed a siRNA specific for PTBP1 to investigate its effects on GC cell viability. AGS and HGC-27 cells were treated with si-PTBP1, and the efficiency of PTBP1 silencing was confirmed using RT–qPCR and western blot (Fig. 1C, D). CCK8 and colony formation assays showed that inhibition of PTBP1 significantly suppressed the viability of GC cells (Fig. 1E, F). We then aimed to elucidate the molecular mechanism underlying the anti-tumor effects of si-PTBP1. Given that PTBP1 is an important RNA-binding protein capable of binding to and regulating mRNA expression, we performed RIP sequencing in SGC-7901 cells to identify 13363 mRNAs potentially bound by PTBP1. Subsequent Gene Ontology (GO) enrichment analysis revealed that autophagy pathway was the most significantly enriched gene set (Fig. 1G). Furthermore, we confirmed a positive correlation between PTBP1 and the expression of autophagy related genes (*ATG10, ATG13, ATG14, LC3*) based on TCGA data (Fig. 1H). These findings suggest that PTBP1 may be involved in regulating autophagy in GC cells. Consequently, our results indicate that interference with PTBP1 significantly inhibits GC cell viability, potentially through the regulation of autophagy.

### Inhibition of PTBP1 contributes to autophagy flux blockage and accumulation of autophagosomes in GC cells

**Fig. 2:**
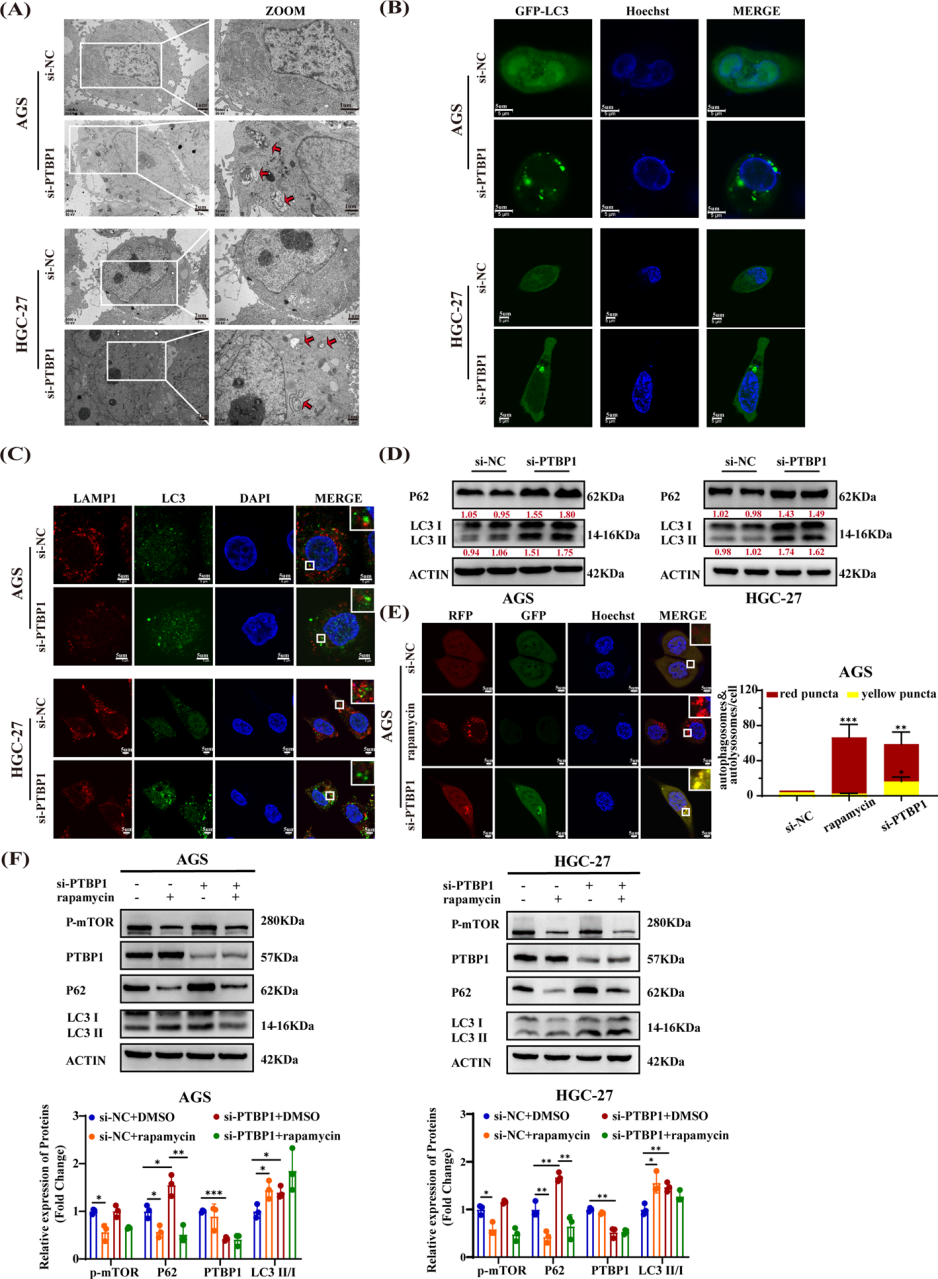
PTBP1 Knockdown leads to an excessive accumulation of autophagosomes, blocking autophagy flux in GC cells. **A** Transmission electron microscopy (TEM) images showing endogenous autophagic microstructures in PTBP1-silenced and control cells. Red arrows indicated autophagosomes or autolysosomes. Scale bars, 1 or 2 μm. **B** AGS and HGC-27 cells were first transfected with GFP-LC3 for 24 h and subsequently transfected with either si-NC or si-PTBP1 for an additional 48 h. Formation of LC3 puncta (green) was detected using a laser confocal microscope, with Hoechst 33342 (blue) staining for nuclei. Scale bar, 5 μm. **C** After stable silencing of PTBP1, the colocalization of LC3B (green) and LAMP1 (red) was assessed. Hoechst 33342 (blue) was used to stain the nuclei, and images were captured using a laser confocal microscope. The white rectangle represents a partial enlargement. Scale bar, 5 μm. **D** Western blot was used to detect the expression levels of P62 and LC3 in PTBP1-silenced and control cells. β-actin served as a control, and densitometric values of band intensity were indicated by red numbers. **E** PTBP1-silenced and control cells were exposed to an mRFP-GFP-LC3 lentivirus. The number of LC3 puncta (yellow for autophagosomes, red for autolysosomes) was quantified. Hoechst 33342 (blue) was used to stain the nuclei, and images were captured using a laser confocal microscope. Scale bar, 5 μm. Data are presented as mean ± SD. Statistical analysis was performed using an unpaired student’s *t*-test, with *n* > 3. *P < 0.05; **P < 0.01; ***P < 0.001. **F** Western blot was used to determine the protein expression levels of P-mTOR, PTBP1, P62, and LC3. β-actin served as a control. PTBP1-silenced and control cells were treated with either DMSO or rapamycin (AGS, 50 nM; HGC-27, 250 nM) for 24 h. Data were presented as mean ± SD, with statistical analysis conducted using an unpaired student’s *t*-test, *n* > 3. *P < 0.05; **P < 0.01; ***P < 0.001

To ascertain the role of PTBP1 in regulating autophagy in GC cells, we knocked down PTBP1 expression in AGS and HGC-27 cell lines and analyzed several autophagy markers. TEM revealed a frequent formation of autophagic vacuoles, indicated by the red arrow, in PTBP1-silenced cells (Fig. 2A). Additionally, immunofluorescence analysis demonstrated a marked increase in LC3 puncta structure in AGS and HGC-27 cells stably expressing GFP-LC3 following PTBP1 inhibition, suggesting an augmentation in autophagosomes (Fig. 2B). Typically, autophagy is accompanied by a concurrent increase in autophagosomes and lysosomes, as well as their colocalization. Therefore, we investigated the colocalization of LC3 with LAMP1, a lysosomal marker, in AGS and HGC-27 cells treated with PTBP1 interference. As illustrated in Fig. 2C, si-PTBP1 resulted in the increase in LC3 fluorescence (green points) but a decrease of LAMP1 fluorescence (red points), with minimal LC3-LAMP1 colocalization observed. This finding indicated that si-PTBP1 impeded fusion of autophagosomes and lysosomes, also known as disrupting the autophagic flux and leading to an abnormal increase in autophagosomes. Autophagic flux, the process where autophagosomes fuse with lysosomes to degrade their contents, was investigated to assess the impact of PTBP1 interference. Western blot experiments showed that silencing PTBP1 concomitantly increased the protein levels of LC3-II/ LC3-I and P62, a well-known autophagic substrate. (Fig. 2D). To further evaluate autophagy flux, AGS cells were transfected with a GFP-mRFP-LC3 lentivirus and treated with either si-NC (negative control), rapamycin (positive control for autophagy), or si-PTBP1 (Fig. 2E). In the si-NC group, diffuse red/green fluorescence was observed, indicating baseline autophagy levels. Rapamycin stimulation led to an increase in red puncta (autolysosomes), signifying an enhancement in autophagy flux. Notably, upon transfection with si-PTBP1, AGS cells exhibited a significant accumulation of yellow puncta (autophagosomes), visually indicating a blockage in autophagy flux. Western blot analysis further corroborated these findings, revealing that rapamycin treatment downregulated p-mTOR and P62 levels while promoting LC3I to LC3II conversion (indicating autophagy activation). Interestingly, rapamycin was able to mitigate the si-PTBP1-induced accumulation of P62, thereby restoring autophagic flux (Fig. 2F). These data collectively demonstrate that silencing PTBP1 blocks autophagic flux, leading to the accumulation of autophagosomes in GC cells.

### PTBP1 inhibits TXNIP expression by binding to and destabilizing TXNIP mRNA

**Fig. 3:**
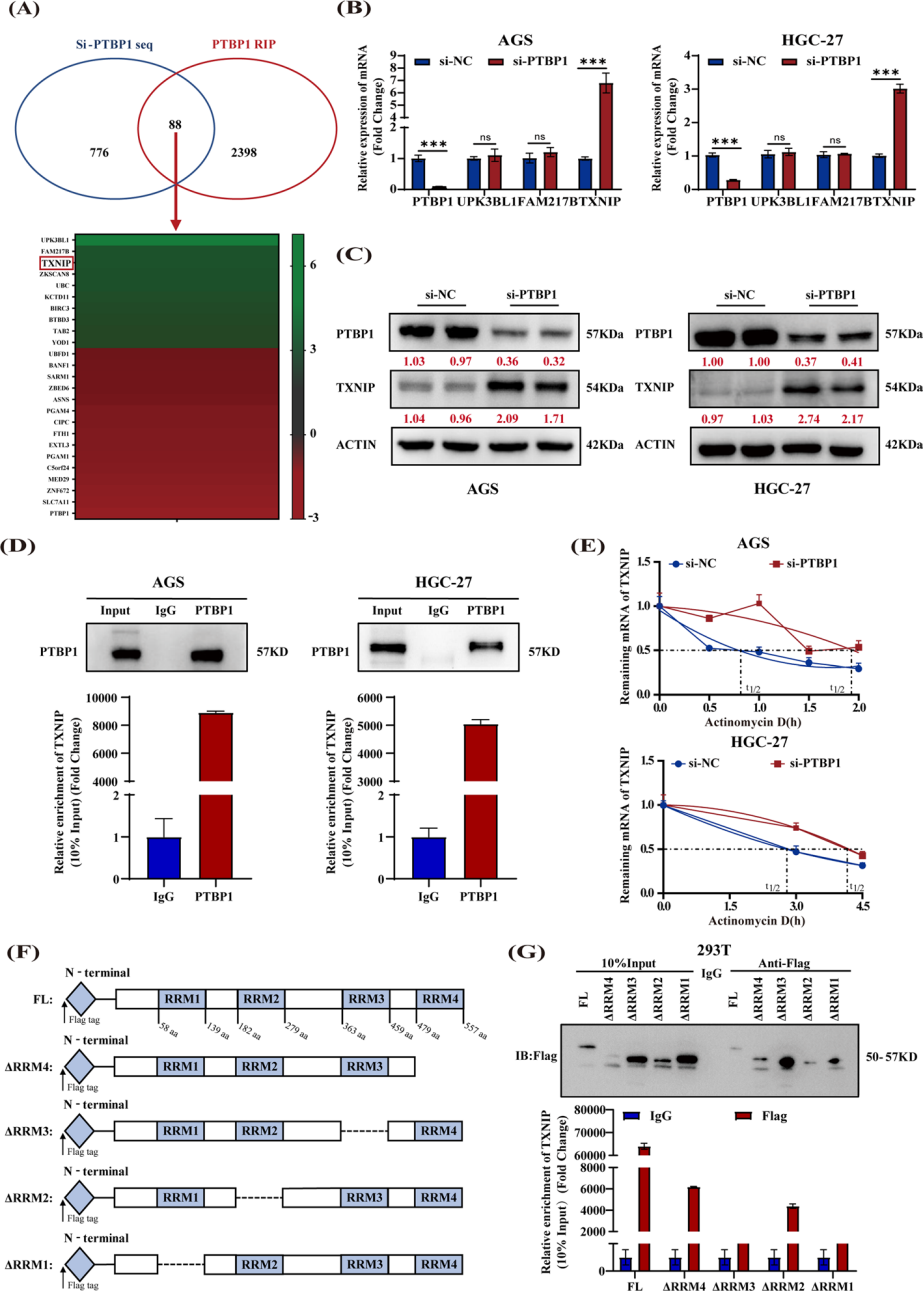
PTBP1 protein promotes *TXNIP* decay through direct binding to its mRNA. **A** Potential target screening strategy for PTBP1. **B** Following PTBP1 knockdown, the expression of *PTBP1*, *UPK3BL1*, *FAM217B*, and *TXNIP* mRNA in AGS and HGC-27 was measured. Data were presented as mean ± SD. Statistical analysis was performed using an unpaired student’s *t*-test, with nonsignificance (ns) > 0.05, ***P < 0.001. **C** After PTBP1 knockdown, the expression of TXNIP and PTBP1 proteins in AGS and HGC-27 cells was assessed, with β-actin serving as a control. Red numbers indicated densitometric values of band intensity. **D** Western blot assays demonstrated the efficiency of the RIPA. The level of TXNIP enriched by PTBP1 was quantified using RT–qPCR. **E** *TXNIP* mRNA stability in AGS and HGC-27 cells was assessed after PTBP1 knockdown and treatment with actinomycin D (AGS, 4 μg/ml; HGC-27, 6 μg/ml).** F** Structural diagram of PTBP1 protein and truncated variants. **G** Full-length or truncated mutants of PTBP1 variants were pulled down using anti-flag and verified by western blot. The expression of enriched TXNIP in different groups was quantified by RT–qPCR

To elucidate the molecular mechanism underlying PTBP1’s impact on autophagy, we analyzed RNA sequencing data from PTBP1 knock-down cells and RIP sequencing data enriched by PTBP1.This intersection of datasets, comprising 361 downregulated and 504 upregulated genes with a RIP sequencing peak score greater than 10 (Supporting Information Table S5), yielded 88 candidate genes. Among these, *UPK3BL1*, *FAM211B*, and *TXNIP* emerged as the top three candidates based on their binding scores (Fig. 3A). Subsequent RT–qPCR analysis revealed that, while *UPK3BL1* and *FAM211B* showed no significant changes, *TXNIP* mRNA was significantly upregulated in GC cells treated with si-PTBP1 (Fig. 3B). Consistent with this finding, western blot analysis confirmed an increase in TXNIP protein levels upon PTBP1 silencing (Fig. 3C). Further, the RIP–PCR experiment showed that PTBP1 highly enriched *TXNIP* mRNA in AGS and HGC-27 cells, indicating a direct binding interaction (Fig. 3D). Moreover, an actinomycin D experiment revealed that PTBP1 silencing extended the half-life of *TXNIP* mRNA, suggesting that PTBP1 disrupts the stability of *TXNIP* mRNA (Fig. 3E). Given that PTBP1 contains four RNA recognition motifs (RRMs), we constructed full-length and truncated PTBP1 expression plasmids and found that TXNIP mainly interacted with RRM1 and RRM3 of PTBP1 (Fig. 3F, G). These results collectively indicate that PTBP1 binds and destabilizes the stability of *TXNIP* mRNA, thereby inhibiting its expression.

### TXNIP is a key regulator in autophagy flux blockage and ROS accumulation induced by PTBP1 inhibition in GC cells

**Fig. 4:**
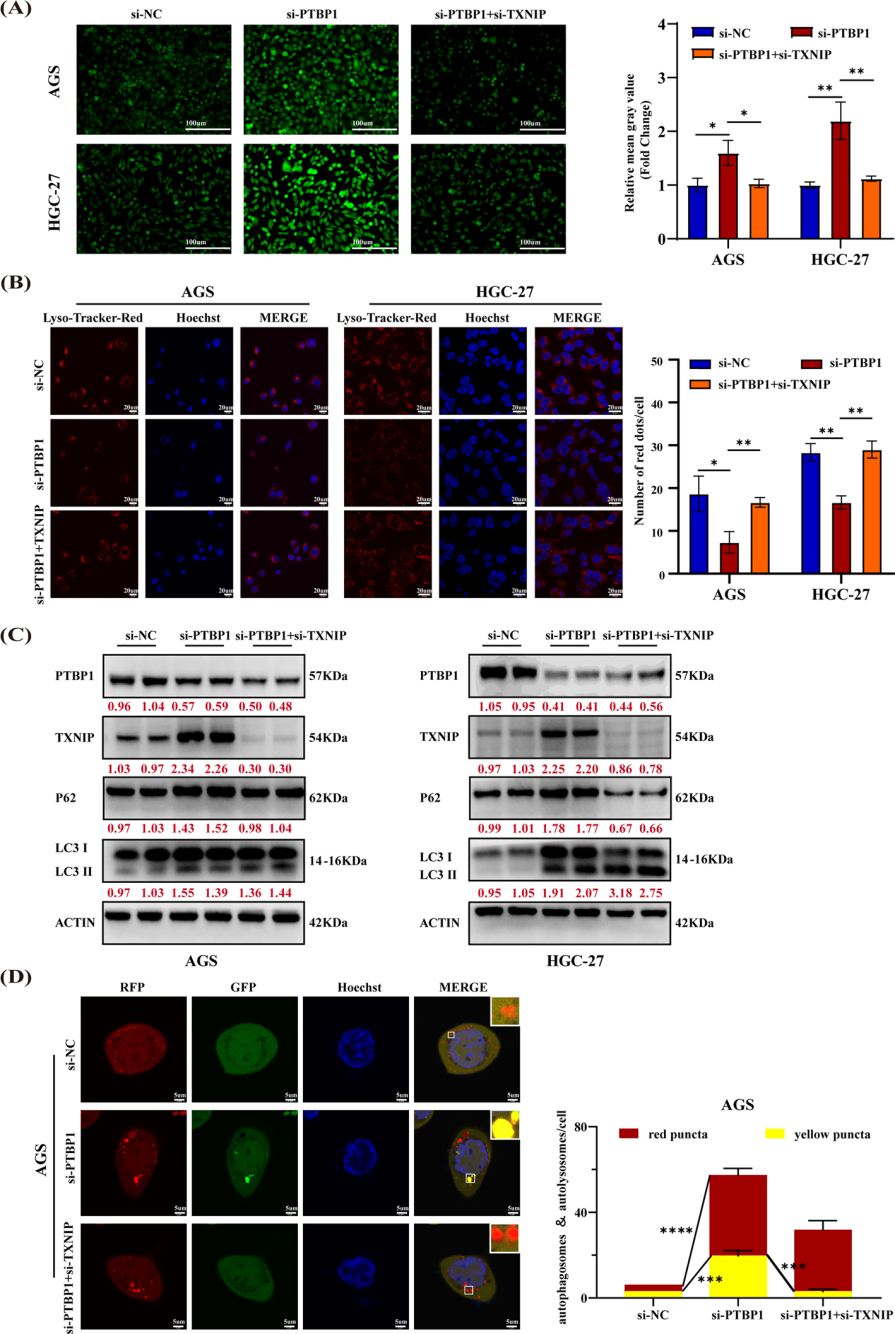
PTBP1 knockdown impedes autophagy flux in GC cells by upregulating TXNIP. **A** Reactive oxygen species (ROS) levels were detected through inverted fluorescence microscope in AGS and HGC-27 cells transfected with si-NC, si-PTBP1, or co-transfected with si-PTBP1 and si-TXNIP. Quantification of the mean gray value was presented as mean ± SD, with statistical analysis conducted using an unpaired student’s *t*-test, *n* = 3. *P < 0.05; **P < 0.01. **B** Lyso-Tracker Red was used to assess lysosomal abundance. Quantification of lysosome numbers was presented as mean ± SD, with statistical analysis conducted using an unpaired student’s *t*-test, *n* = 3. *P < 0.05; **P < 0.01. **C** Protein levels of P62, PTBP1, TXNIP, and LC3 were assessed in AGS and HGC-27 cells transfected with si-NC, si-PTBP1, or co-transfected with si-PTBP1 and si-TXNIP. β-actin served as a control. Red numbers indicated densitometric values of band intensity. **D** Autophagy flux was assessed in mGFP-RFP-LC3-stably expressed AGS cells transfected with si-NC, si-PTBP1, or co-transfected with si-PTBP1 and si-TXNIP. The number of LC3 puncta (yellow for autophagosomes, red for autolysosomes) was quantified. Hoechst 33342 (blue) was used for nuclear staining, and cells were imaged under a laser confocal microscope. Scale bar, 5 μm. Data were presented as mean ± SD, with statistical analysis conducted using an unpaired student’s *t*-test, *n* = 3. ***P < 0.001

Given that TXNIP promotes ROS production, which may ultimately block autophagic flux by inducing lysosomal damage [29], we investigated the impact of the PTBP1–TXNIP axis on ROS levels using DCFH-DA staining. Fluorescence analysis indicated that si-PTBP1 led to an increase in fluorescence intensity in AGS and HGC-27 cells, suggesting ROS accumulation. However, co-silencing of PTBP1 and TXNIP weakened the elevation in ROS levels (Fig. 4A). To assess lysosomal damage, we performed immunofluorescence analysis using the Lyso-Tracker Red probe. As depicted in Fig. 4B, si-PTBP1 weakened the red fluorescence, indicating lysosomal dysfunction. Nevertheless, concurrent silencing of PTBP1 and TXNIP rescued the red fluorescence. Additionally, western blot analysis showed that PTBP1 knockdown increased the protein levels of P62 and LC3-II/LC3-I, while cosilencing of PTBP1 and TXNIP partially restored P62 levels (Fig. 4C). To further validate these findings, we knocked down PTBP1 and TXNIP simultaneously in AGS cells infected with GFP-mRFP-LC3 lentivirus. Immunofluorescence showed that cosilencing of PTBP1 and TXNIP counteracted the increase in yellow puncta (autophagosomes) induced by PTBP1 knockdown (Fig. 4D). Collectively, these results suggest that TXNIP plays a pivotal role in the blockage of autophagic flux induced by PTBP1 knockdown in GC cells.

### PTBP1 inhibition exerts anti-tumor effect in a TXNIP dependent manner

**Fig. 5:**
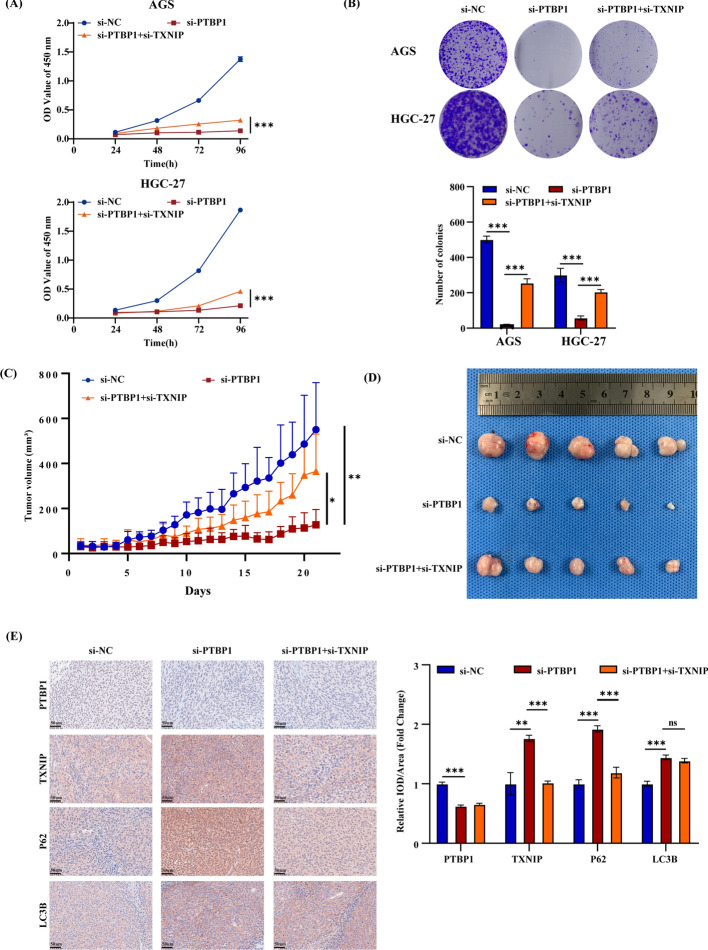
PTBP1 knockdown induces antitumor effects in a TXNIP-dependent manner. **A**,**B** CCK-8 assay (**A**) and colony formation (**B**) of AGS and HGC-27 cells were assessed upon transfection with si-NC, si-PTBP1, or co-transfection with si-PTBP1 and si-TXNIP, as indicated. Data are presented as mean ± SD with statistical analysis conducted using an unpaired student’s *t*-test, *n* = 3. ***P < 0.001. **C**,**D** Xenograft tumors derived from HGC-27 cells were injected with si-PTBP1 lentivirus, cholesterol-modified si-PTBP1, or cholesterol-modified si-TXNIP. Growth curves (**C**) and tumor images (**D**) were obtained from these xenograft tumors. **E** Representative immunohistochemical (IHC) images depicting the expression of PTBP1, TXNIP, P62, and LC3B in xenografted tumor tissues. Scale bar, 50 μm. Data are presented as mean ± SD, with statistical analysis conducted using an unpaired student’s *t*-test, *n* = 3. ns > 0.05, **P < 0.01, ***P < 0.001

To examine whether the diminished viability of GC cells resulting from si-PTBP1 was mediated through TXNIP expression modulation and autophagic flux inhibition, we conducted in vitro functional recovery experiments. The CCK8 and colony formation assays revealed a significant suppression of GC cell viability by si-PTBP1 compared with the control group. Interestingly, the combination of si-PTBP1 and si-TXNIP partially restored cell viability (Fig. 5A, B). To further validate these findings, we utilized HGC-27 cells to establish a nude mouse xenograft model. The nude mice were then randomly assigned to three groups: the control group, cholesterol-modified si-PTBP1 treatment group, and the combination group treated with both cholesterol-modified si-PTBP1 and si-TXNIP. Our results showed that silencing PTBP1 alone significantly reduced tumor growth rate and size compared with the control group. However, inhibiting TXNIP expression partially reversed the antitumor effect of si-PTBP1 (Fig. 5C, D). Immunohistochemical results of xenograft tumor showed that PTBP1 knockdown increased LC3B and P62 expression, suggesting autophagic flux blockade. The combined knockdown of PTBP1 and TXNIP partially alleviated the P62 expression increase induced by PTBP1 interference, indicating a partial relief of autophagic flux blockade (Fig. 5E). Collectedly, these results indicate that the decrease in GC cell viability caused by si-PTBP1 mediated through autophagic flux inhibition in a TXNIP dependent manner.

### Si-PTBP1 enhances anti-tumor effect of chloroquine through blocking autophagy flux in vitro and in vivo

**Fig. 6:**
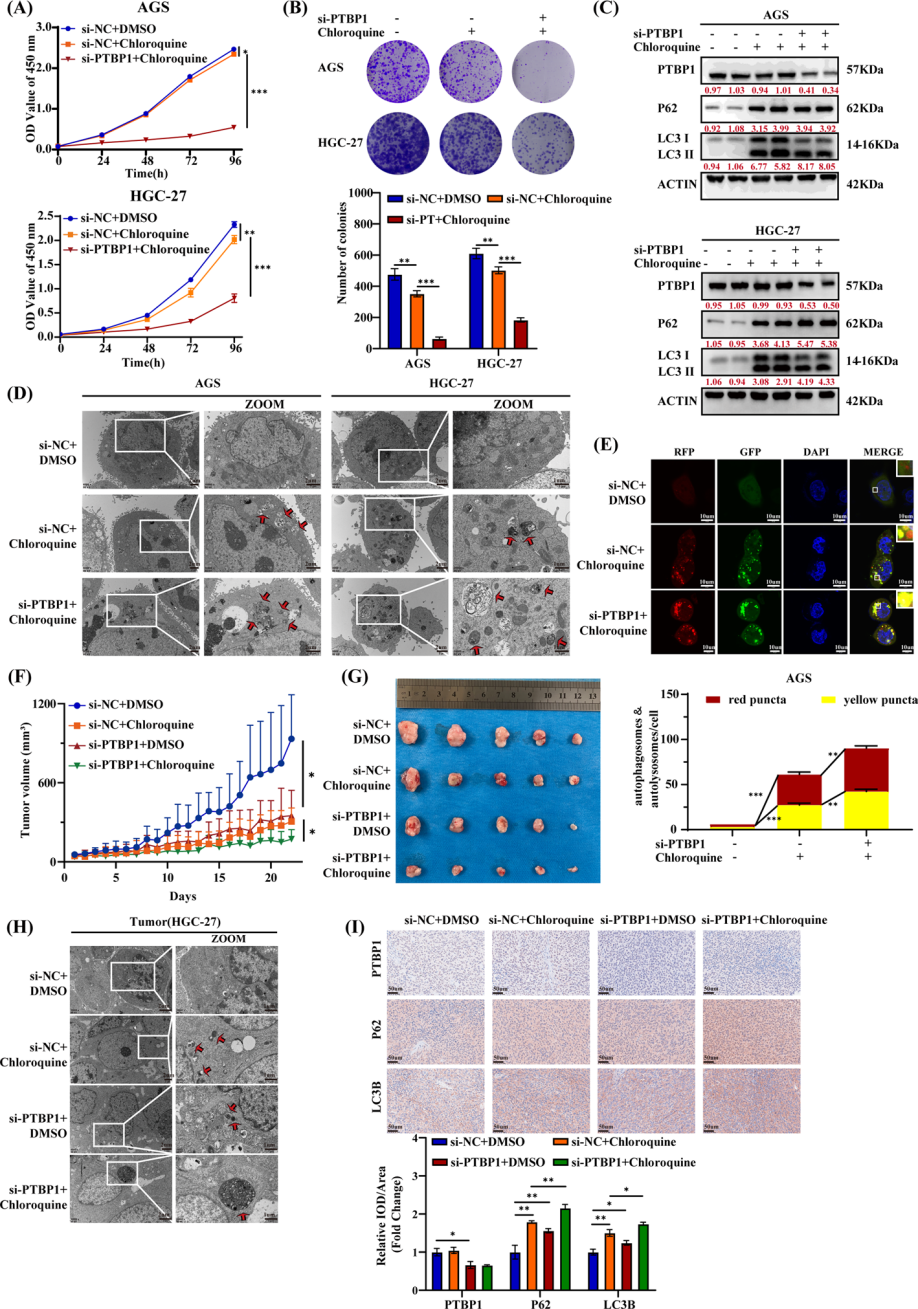
PTBP1 knockdown enhances chloroquine’s antitumor effects both in vitro and in vivo. **A**,**B** CCK-8 assay (**A**) and colony formation (**B**) of AGS and HGC-27 cells were assessed upon transfection with si-NC or si-PTBP1 as indicated. Cells were treated with DMSO or chloroquine (AGS, 20 μM; HGC-27, 100 μM) for 24 h. **C** Protein expression levels of P62, PTBP1, TXNIP, and LC3 were assessed in AGS and HGC-27 cells transfected with si-NC or si-PTBP1. β-actin served as a control. Red numbers indicated densitometric values of band intensity. Cells were treated with DMSO or chloroquine (AGS, 20 μM; HGC-27, 100 μM) for 24 h. **D** Transmission electron microscopy (TEM) images depicting endogenous autophagic structures in PTBP1-silenced and control cells. Cells were treated with DMSO or chloroquine (AGS, 20 μM; HGC-27, 100 μM) for 24 h. Red arrows indicate autophagosomes or autolysosomes. Scale bars, 1 or 2 μm. **E** Autophagy flux was assessed in AGS cells stably expressing mGFP-RFP-LC3 and transfected with si-NC or si-PTBP1. Cells were treated with DMSO or chloroquine (20 uM) for 24 h. LC3 puncta were quantified, representing autophagosomes (yellow) and autolysosomes (red). Hoechst 33342 (blue) stained the nuclei. Scale bar, 10 μm. **F**,**G** Xenograft tumors derived from HGC-27 cells were injected with si-PTBP1 lentivirus, cholesterol-modified si-PTBP1, or chloroquine (40 mg/kg). Growth curves (**F**) and tumor images (**G**) were obtained from these xenograft tumors. **H** Representative TEM images evaluating endogenous autophagic structures in xenografted tumor tissues. Red arrows indicated autophagosomes or autolysosomes. Scale bars, 1 or 2 μm. **J** Representative immunohistochemical (IHC) images depicting the expression of PTBP1, P62, and LC3B in xenografted tumor tissues. Scale bar, 50 μm. Data were presented as mean ± SD, with statistical analysis conducted using an unpaired student’s *t*-test, *n* = 3. *P < 0.05, **P < 0.01

The aforementioned properties of si-PTBP1 in inhibiting autophagic flux and promoting autophagosome accumulation suggest its potential as a therapeutic agent for GC. While autophagic flux inhibitors like chloroquine have demonstrated effectiveness against cancers in clinical trials, their routine application in practice is still a long way off. In this study, we compared the antitumor effects of chloroquine and PTBP1 inhibition-based combination therapy for GC. Our validation revealed that chloroquine treatment significantly elevated LC3 II/I and P62 levels in HGC-27 and AGS cells, resembling the effects of si-PTBP1 (Figure S2). However, chloroquine alone did not significantly inhibit the growth of AGS and HGC-27 cells. In contrast, the combination of chloroquine and si-PTBP1 markedly suppressed the vitality and growth of GC cells (Fig. 6A, B). Moreover, compared with chloroquine alone, the combination therapy with si-PTBP1 further enhanced the accumulation of LC3 II/I and P62 (Fig. 6C). TEM analysis showed that chloroquine increased autophagosome formation in AGS and HGC-27 cells, and the combination therapy group exhibited an even higher number of autophagosomes (Fig. 6D). To further assess autophagic flux, a tandem fluorescent-tagged LC3 reporter system (GFP-mRFP-LC3) was used. Confocal microscope revealed a higher abundance of yellow puncta in the combined therapy group (chloroquine + si-PTBP1) compared with cells treated with chloroquine alone, indicating an exacerbation of autophagic flux blockage following PTBP1 inhibition (Fig. 6E). To determine the in vivo efficacy of this combined approach, HGC-27 cell xenografts were implanted in nude mice. After 1 week, the mice were randomly assigned to four groups: control group, chloroquine alone, PTBP1 silencing alone, and chloroquine combined with PTBP1 silencing. Both chloroquine and PTBP1 silencing alone reduced tumor growth rate and volume compared with the control. Impressively, the combination of chloroquine and PTBP1 silencing significantly enhanced the antitumor effect, exhibiting the slowest tumor growth rate and the smallest tumor volume (Fig. 6F, G). TEM and IHC analyses of tumor tissues from the mouse xenograft models revealed that the combination therapy increased autophagosome formation and intensified the accumulation of LC3B and P62 (Fig. 6H, I). These results indicate that PTBP1 inhibition can enhance the anti-GC effect of chloroquine in vivo and in vitro by blocking autophagic flux.

## Discussion

PTBP1, a heterogeneous ribonucleoprotein, is widely expressed in various tissues and cells, including embryonic stem cells. As an RNA-binding protein, PTBP1 performs multiple molecular functions, such as regulating alternative splicing (AS) [30], controlling mRNA stability [19], and determining mRNA localization [18]. Recent research has highlighted the pivotal role of PTBP1 in the progression of various cancers [30–32], including GC. For instance, PTBP1 can interact with long noncoding RNA (lncRNA) CCAT1, inhibiting ubiquitin-mediated degradation of PTBP1, thus enhancing glycolysis and promoting GC progression [33]. Our previous study [19] demonstrated that PTBP1 interacts with and stabilizes *PGK1* mRNA, thereby promoting GC metastasis. Additionally, PTBP1 has been reported to drive c-Myc-dependent GC progression and stemness [34]. In this study, we observed high expression of PTBP1 in GC, and silencing PTBP1 significantly inhibited cell viability and colony formation, which was attributed to the regulation of autophagy in GC cells by si-PTBP1. Interestingly, our findings revealed that knockdown of PTBP1 led to an excessive accumulation of autophagosomes, as evidenced by TEM, immunofluorescence, and western blot experiments.

Autophagy, a catabolic process, comprises several distinct stages: initiation, nucleation, maturation, fusion, and degradation. In this process, class III PI3K complexes are responsible for membranes generation, while proteins such as VPS34, beclin-1, AMBRA1, and mATG9 regulate the formation of autophagic structures [35]. Additionally, the maturation of autophagosomes involves the accumulation of specific proteins at membrane sites, facilitated by the binding of additional ATGs [35]. Once formed, autophagosomes migrate to lysosomes for degradation, a pivotal step in maintaining the cell’s autophagic flow and overall homeostasis. Disruptions in lysosomal function or autophagic flow can result in the accumulation of autophagosomes, which has been implicated in various human diseases [36, 37]. In our study, we monitored the levels of both LC3 and P62 and assessed autophagic flow by examining the colocalization of LAMP1 and LC3, along with the utilization of a double-labeled adenovirus vector expressing mRFP-GFP-LC3. Additionally, we verified the regulation of autophagy in GC cells using a combination of autophagy activator rapamycin or autophagy inhibitor chloroquine with PTBP1 interference. Our findings demonstrate that interference with PTBP1 inhibits autophagic flux in GC cells. The relationship between PTBP1 and autophagy appears to vary among different tumors. For instance, Wang et al. observed that PTBP1 depletion significantly inhibited autophagic flux in RKO cells, which aligns with our current findings [38]. However, previous studies have reported contrasting results, indicating that PTBP1 interference promoted autophagy in breast cancer [39], olfactory mucosa mesenchymal stem cells [40], and GC [41]. Notably, these studies did not investigate the effect of PTBP1 interference on autophagic flux. Given that the accumulation of autophagosomes can be attributed to either an increase in their formation or a blockage in autophagic flux, further investigation is necessary to determine whether si-PTBP1 leads to the initiation of autophagosome formation in the present study.

Next, we delved into the downstream molecular mechanism underlying how PTBP1 impacts autophagic flux. Previous studies have shown that PTBP1 modulates the expression of downstream target genes through diverse mechanisms, particularly in regulating mRNA stability. For example, PTBP1 has been found to enhance the stabilization of a series of mRNAs, including *PGK1* [19], *CFTR* [42], and *PIK3R5* [43]. Conversely, in certain cases, PTBP1 binds to target mRNAs and participates in their decay [44]. For instance, PTBP1 can directly interact with the 5′-UTR of *AXL* mRNA in vitro and in vivo, thereby reducing its stability [45]. Furthermore, PTBP1 has been reported to induce cholestatic liver injury by interacting with *Shp* mRNA and thus inhibiting its stability. In our study, by intersecting the transcriptome sequencing data of si-PTBP1-treated cells with PTBP1 RIP sequencing data, we demonstrated that PTBP1 suppressed *TXNIP* mRNAs stability by directly binding to *TXNIP* mRNA. Specifically, RRM1 and RRM3, which are key domains of the PTBP1 protein, are likely responsible for the interaction with *TXNIP* mRNA.

TXNIP, also referred to as vitamin D3-upregulated protein-1 or thioredoxin (TRX)-binding protein-2, functions as a natural inhibitor of TRX [46]. It plays a pivotal role in regulating cellular redox homeostasis and stimulating the production of ROS in various human diseases [47–49]. ROS, serving as intracellular signaling molecules, are crucial for maintaining redox homeostasis and lysosomal functions [50, 51]. Several studies have demonstrated that TXNIP disrupts lysosomal function. For example, Su et al. [52] demonstrated that TXNIP impairs lysosomes by downregulating the lysosomal membrane protein ATP13A2. In our study, we demonstrated that PTBP1 knockdown impedes autophagic flux in GC cells by upregulating TXNIP. Moreover, PTBP1 inhibition exerts antitumor effect in vitro and in vivo through a TXNIP dependent mechanism. There are currently some analogous findings that corroborate our observations regarding the role of TXNIP in autophagy. For example, Park et al. [53] established a positive correlation between increased TXNIP expression and elevated MAP1LC3B puncta and P62 expression in human nonalcoholic fatty liver disease, providing clinical evidence of the relationship between TXNIP upregulation and autophagy impairment. In addition, Gao et al. [54] found that TXNIP suppressed autophagosome clearance by increasing ROS level in ischemia/reperfusion (I/R) injury. Thus, our study demonstrated that TXNIP could impair lysosome function and inhibit autophagosome clearance through ROS induction. Collectively, these results suggest that PTBP1 regulates autophagy in TXNIP-dependent manner in GC.

## Conclusion

**Fig. 7:**
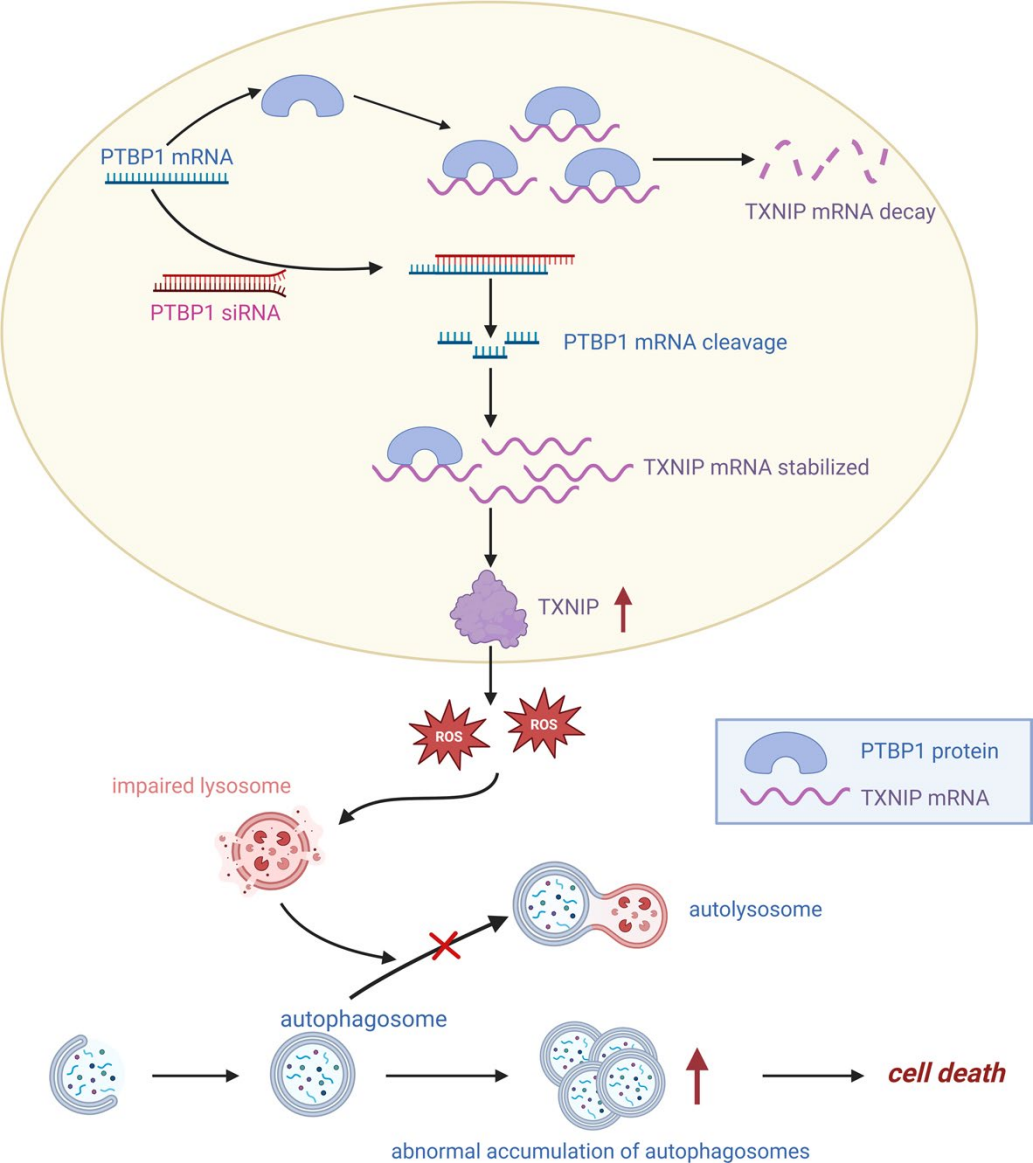
Molecular mechanism diagram. Created with BioRender.com. PTBP1 was highly expressed in GC cells and suppressed *TXNIP* mRNA stability by directly binding to its mRNA. With the knockdown of PTBP1, TXNIP expression was upregulated and thus led to oxidative stress. Excess intracellular ROS led to impaired lysosomal function thereby failing to fuse with autophagosomes to form autolysosomes. Therefore, autophagosomes are over-accumulated in cells, eventually resulting in cell death

In this study, we have validated that interference with PTBP1 significantly impairs the viability of GC cells and curtails tumor growth, both in vitro and in vivo. Mechanistically, interference with PTBP1 leads to the upregulation of TXNIP expression, achieved through the enhancement of *TXNIP* mRNA stability. The elevated TXNIP levels disrupt lysosomal function, resulting in the obstruction of autophagic flux and ultimately triggering cell death in GC cells (Fig. 7).

### Supplementary Information

Supplementary material 1: File S1. The structural maps of the plasmids (GFP-LC3 plasmid, mRFP-GFP-LC3 lentivirus).

Supplementary material 2: File S2. The protein levels of P62 and LC3 in chloroquine-treated and control cells.

Supplementary material 3: File S3. Uncropped blots for figures.

Supplementary material 4: Table S1. Characteristics of patients with GC for PTBP1 measurement; Table S2. Sequences of SiRNAs and primers; Table S3. Differentially expressed genes after silencing PTBP1 in AGS cells; Table S4. Enriched genes from PTBP1 RIP-seq in SGC-7901 cells; Table S5 the overlap between si-PTBP1 RNA-seq and RIP sequencing (peak score > 10).

## Data Availability

The datasets used and analyzed in this paper are available from the corresponding author upon reasonable request.

## Abbreviations

CCK-8: Cell counting Kit-8
DMSO: Dimethyl sulfoxide
GC: Gastric cancer
GEPIA: Gene expression profiling interactive analysis
IgG: Immunoglobulin G
mRNA: Messenger ribonucleic acid
NC: Negative control
OD: Optical density
PBS: Phosphate buffer solution
PBST: Phosphate buffer solution with Tween-20
PCR: Polymerase chain reaction
PTBP1: Polypyrimidine tract-binding protein 1
RBP: RNA binding protein
RIP: RNA Immunoprecipitation
RIP-seq: Ribonucleic acid immunoprecipitation-sequencing
ROS: Reactive oxygen species
RRM: RNA recognition motif
RT–qPCR: Quantitative real-time polymerase chain reaction
shRNA: Small hairpin RNA
siRNA: Small interfering RNA
si-PTBP1 RNA seq: small-interfering PTBP1 RNA sequencing
TCGA: The Cancer Genome Atlas
TXNIP: Thioredoxin-interacting protein
