# Supplementary figures and images for: PTBP1 knockdown impairs autophagy flux and inhibits gastric cancer progression through TXNIP-mediated oxidative stress
Source: Cell Mol Biol Lett. 2024 Aug 17;29:110. doi: 10.1186/s11658-024-00626-1 (PMC11330137; doi:10.1186/s11658-024-00626-1)

A. The structural maps of GFP-LC3 plasmid

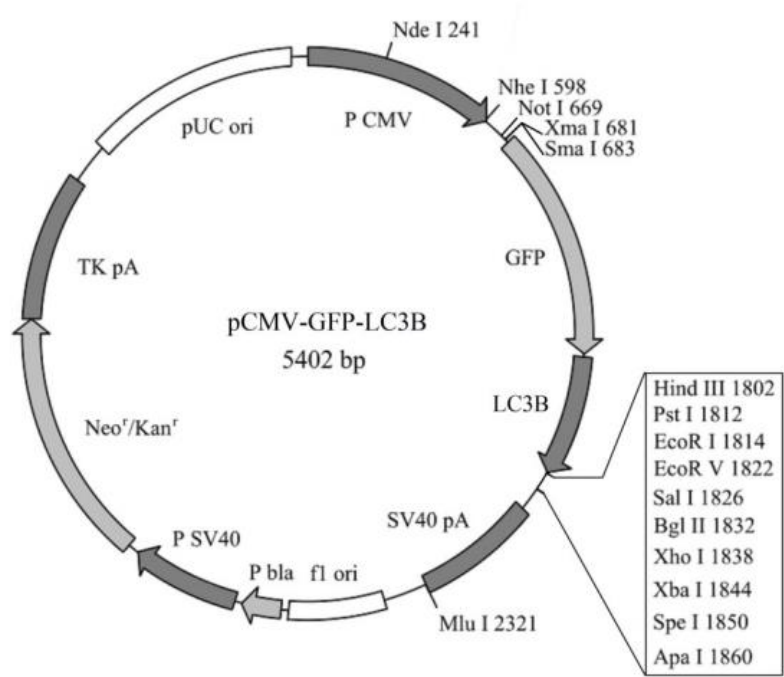

B. The structural maps of mRFP-GFP-LC3 lentivirus

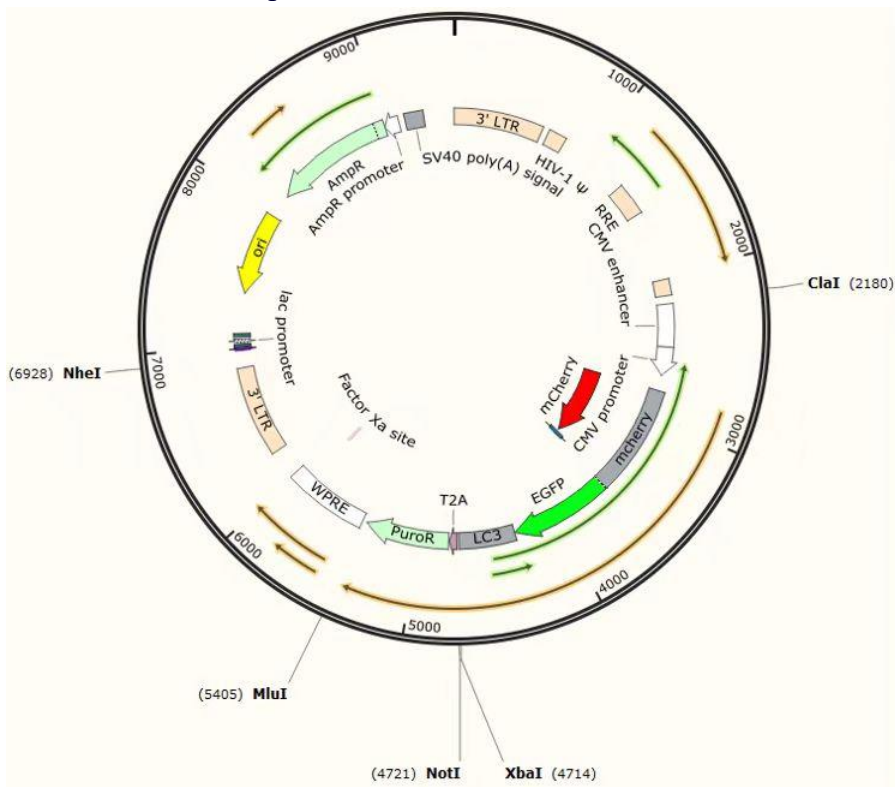

Supplement: Supplementary file 1 — Supplementary material 1: File S1. The structural maps of the plasmids (GFP-LC3 plasmid, mRFP-GFP-LC3 lentivirus). [file 11658_2024_626_MOESM1_ESM.pdf]

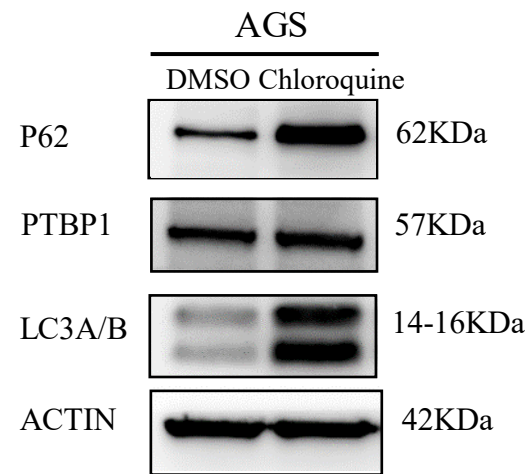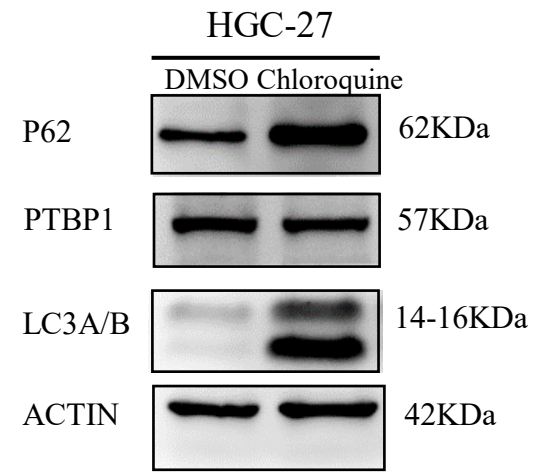

Supplement: Supplementary file 2 — Supplementary material 2: File S2. The protein levels of P62 and LC3 in chloroquine-treated and control cells. [file 11658_2024_626_MOESM2_ESM.pdf]
