# Supplementary material for: PTBP1 knockdown impairs autophagy flux and inhibits gastric cancer progression through TXNIP-mediated oxidative stress
Source: Cell Mol Biol Lett. 2024 Aug 17;29:110. doi: 10.1186/s11658-024-00626-1 (PMC11330137; doi:10.1186/s11658-024-00626-1)

File S2

Uncropped blots for Figure 1B

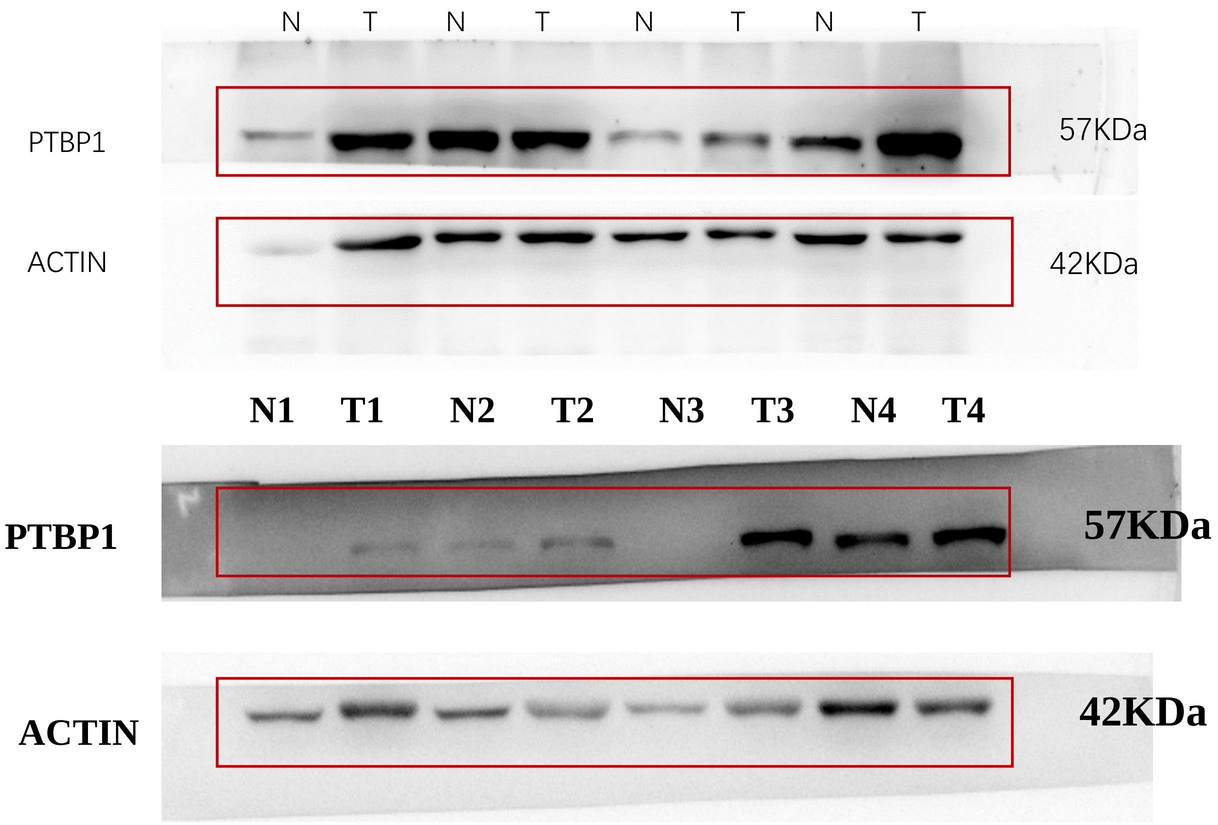

Uncropped blots for Figure 1D

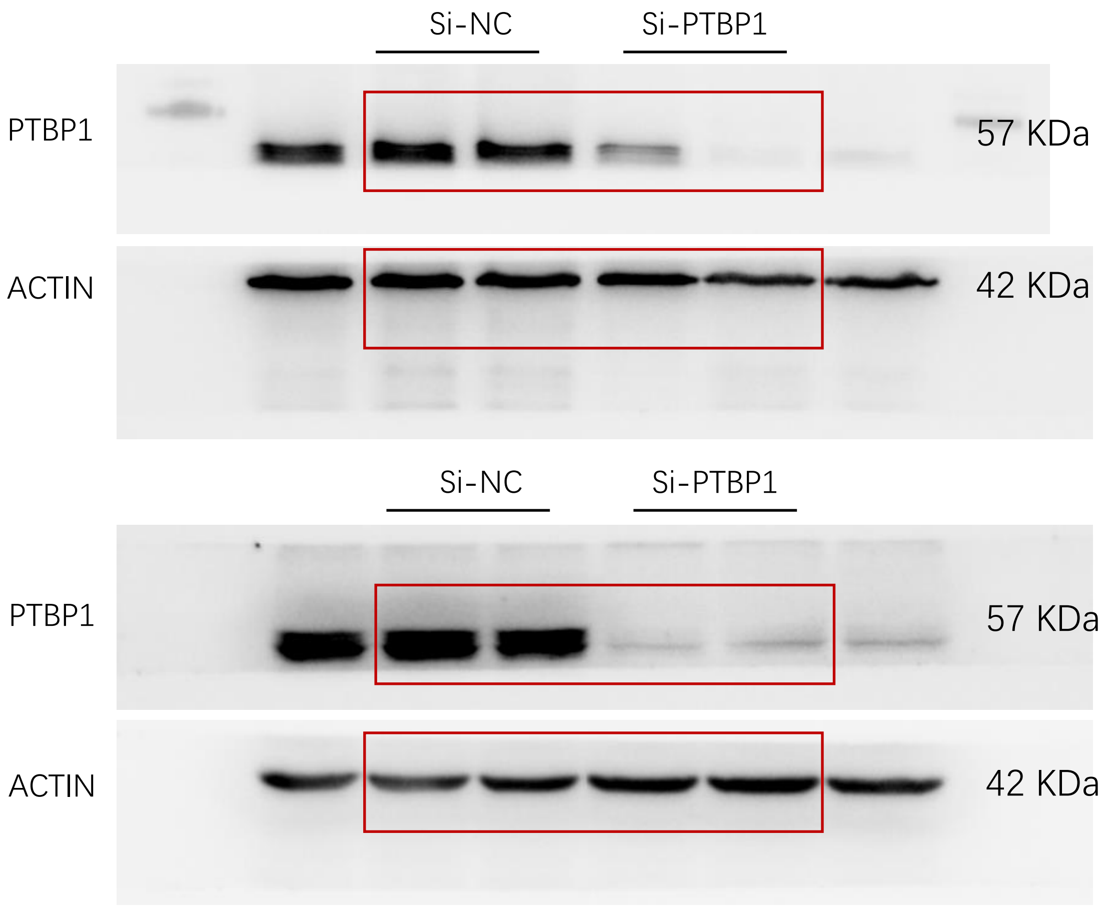

File S2

Uncropped blots for Figure 2D

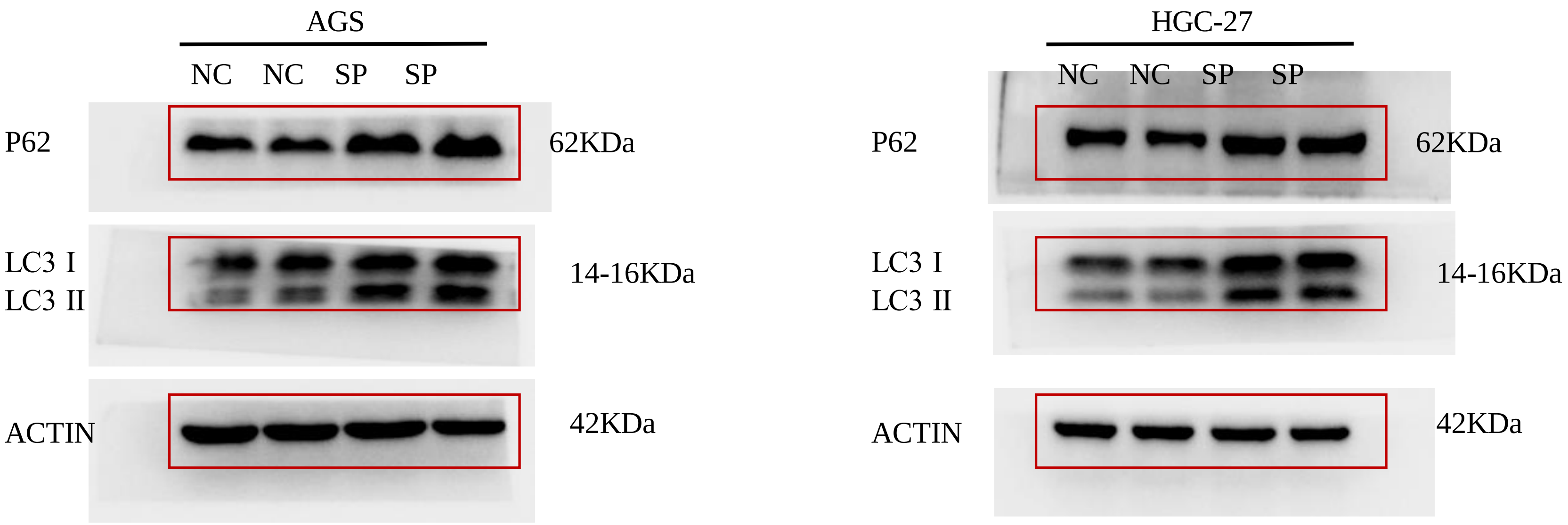

File S2

Uncropped blots for Figure 2F

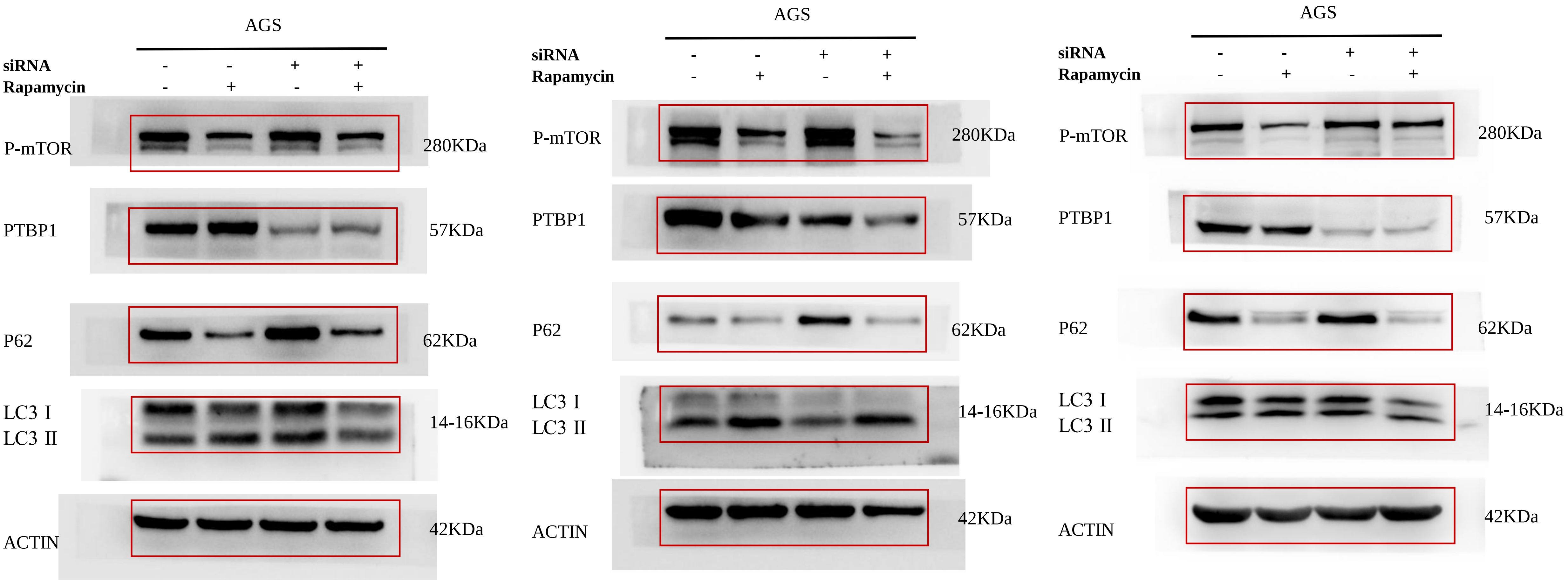

File S2

Uncropped blots for Figure 2F

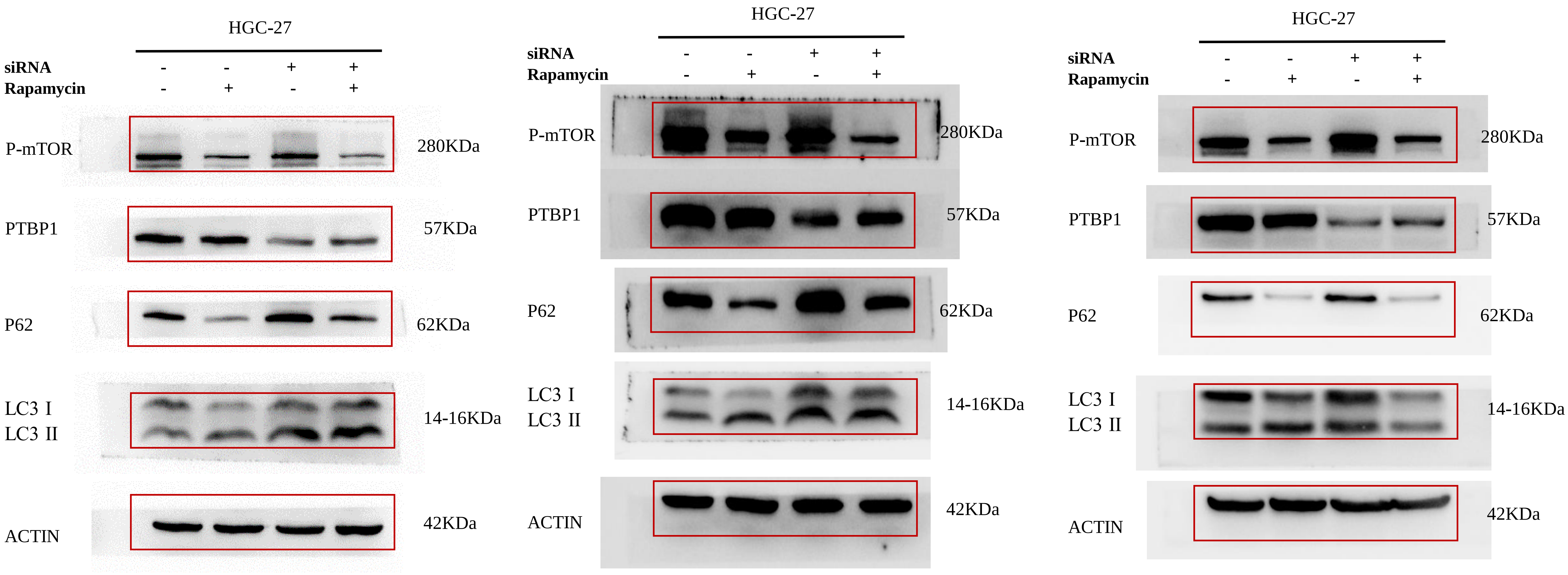

File S2

Uncropped blots for Figure 3C

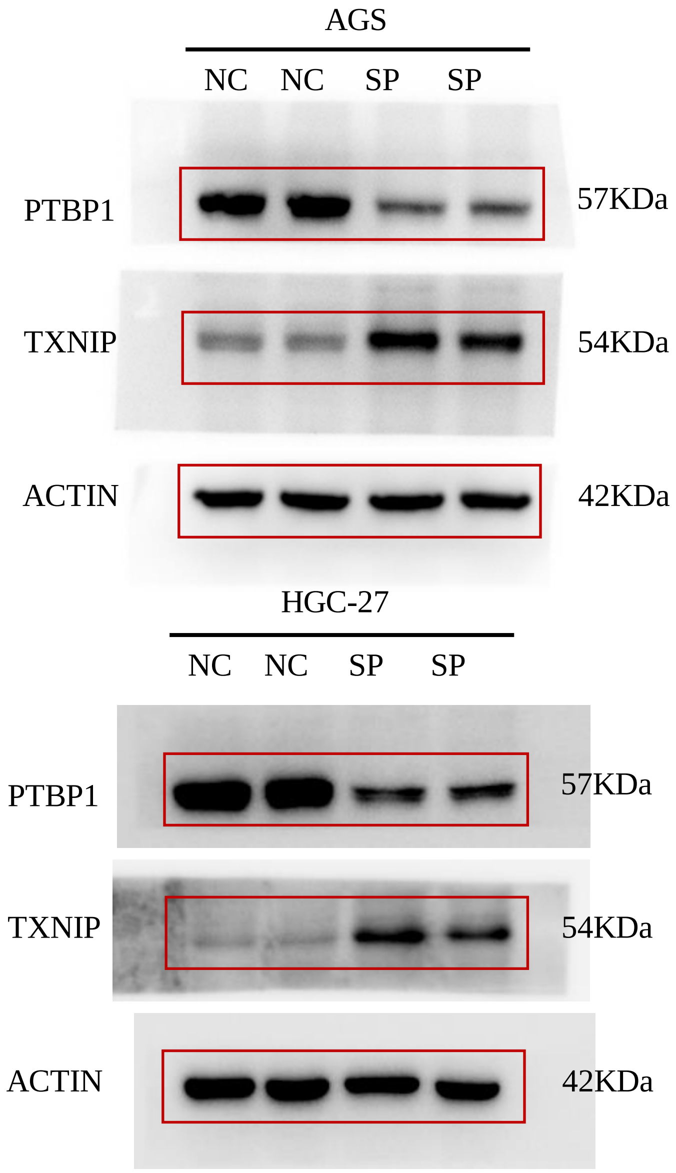

Uncropped blots for Figure 3D

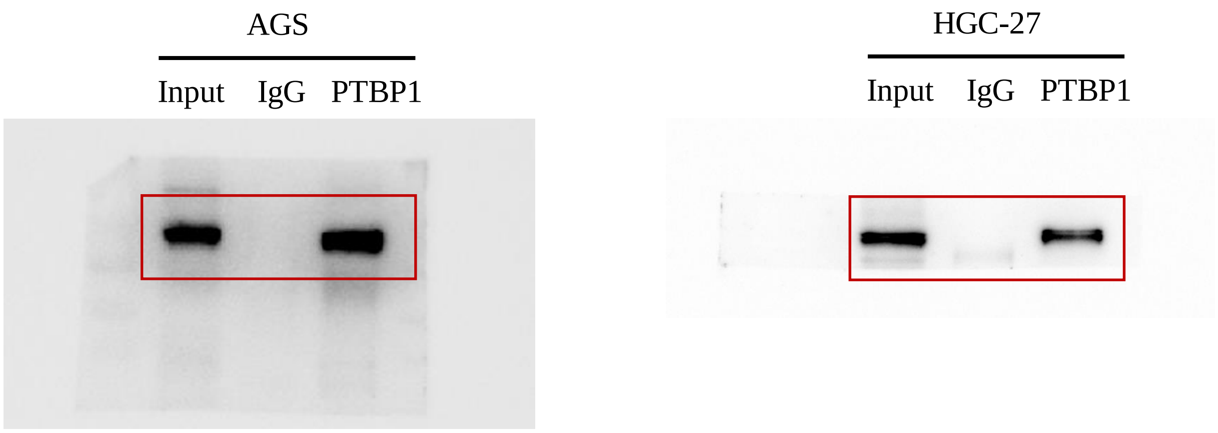

Uncropped blots for Figure 3G

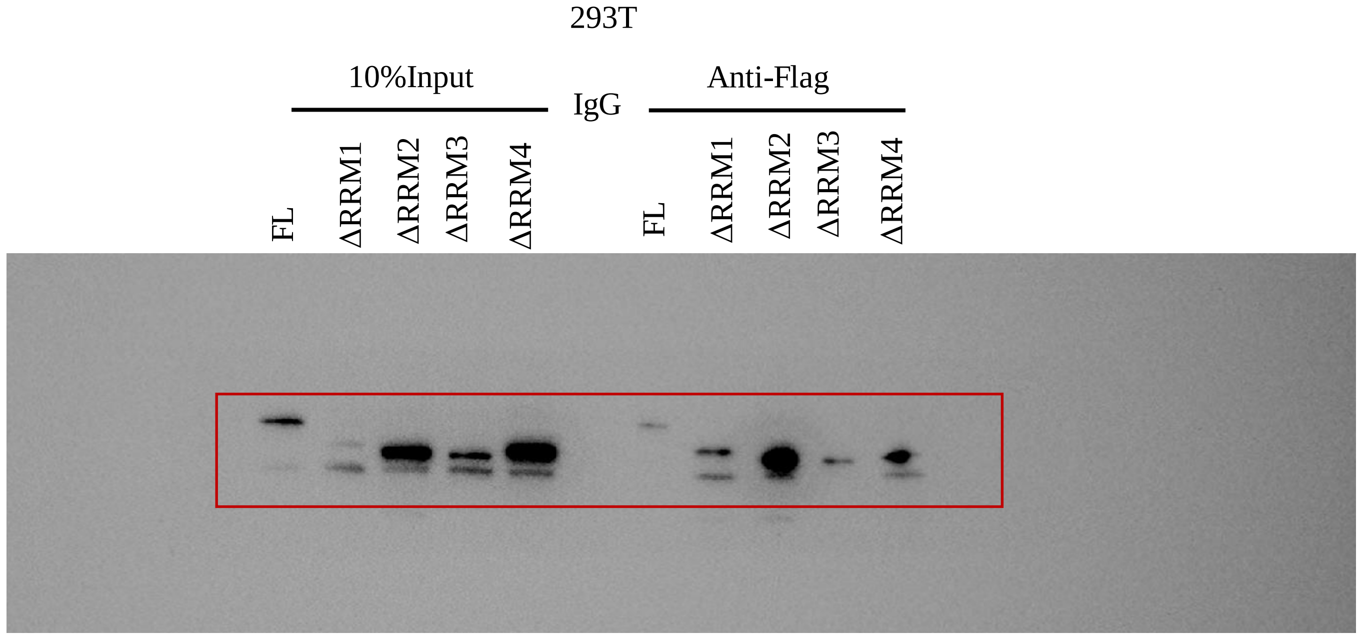

File S2

Uncropped blots for Figure 4C

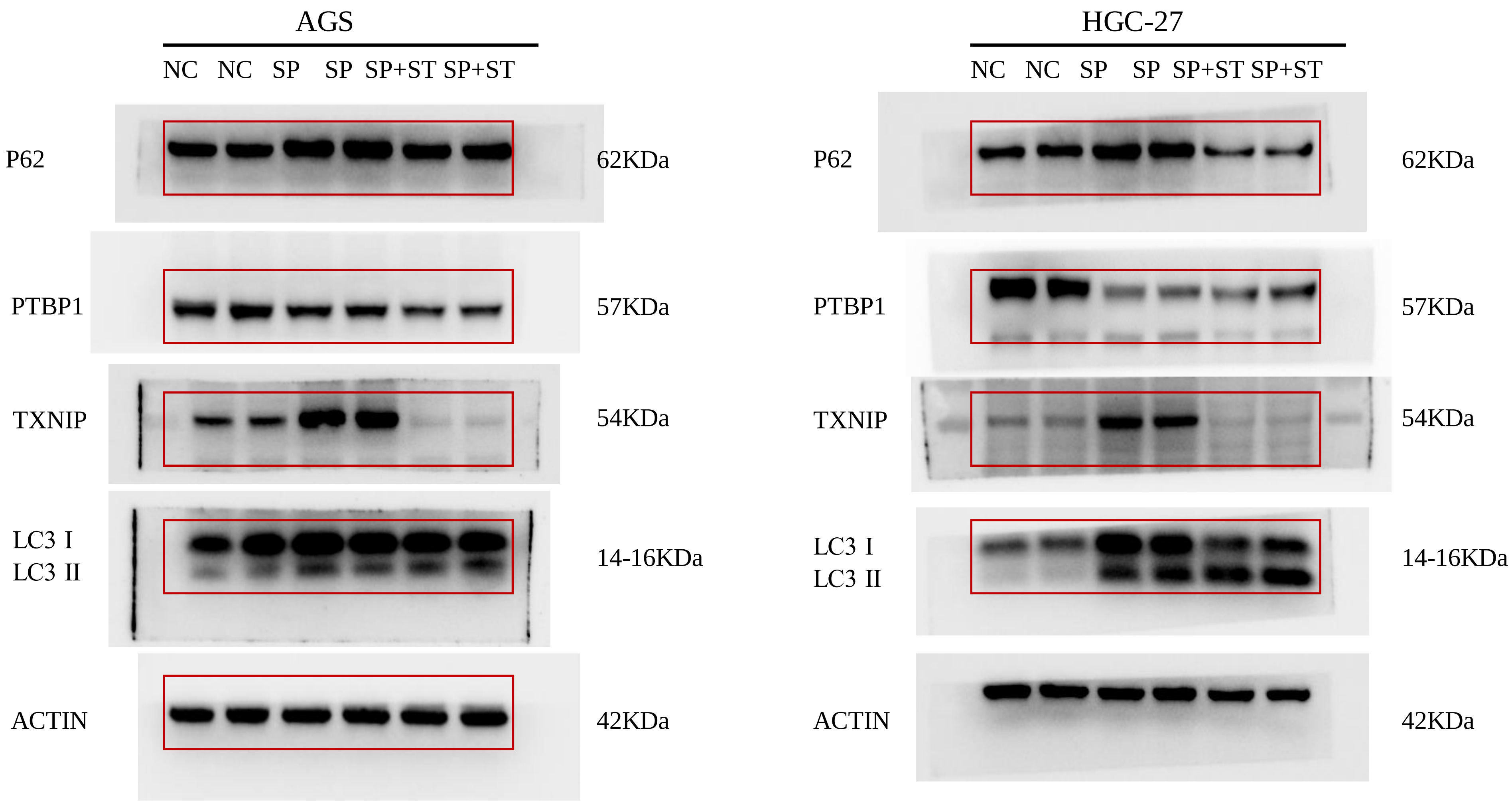

File S2

Uncropped blots for Figure 6C

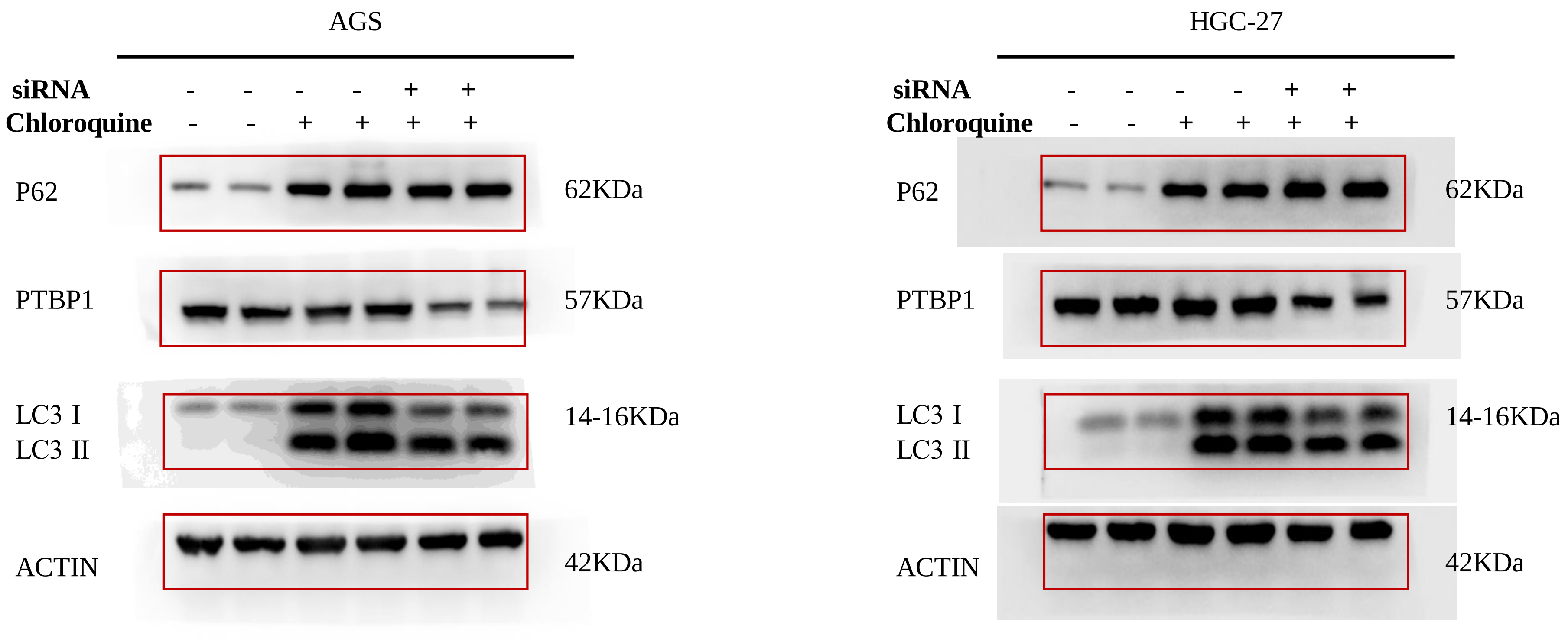

File S2

Uncropped blots for Figure S1

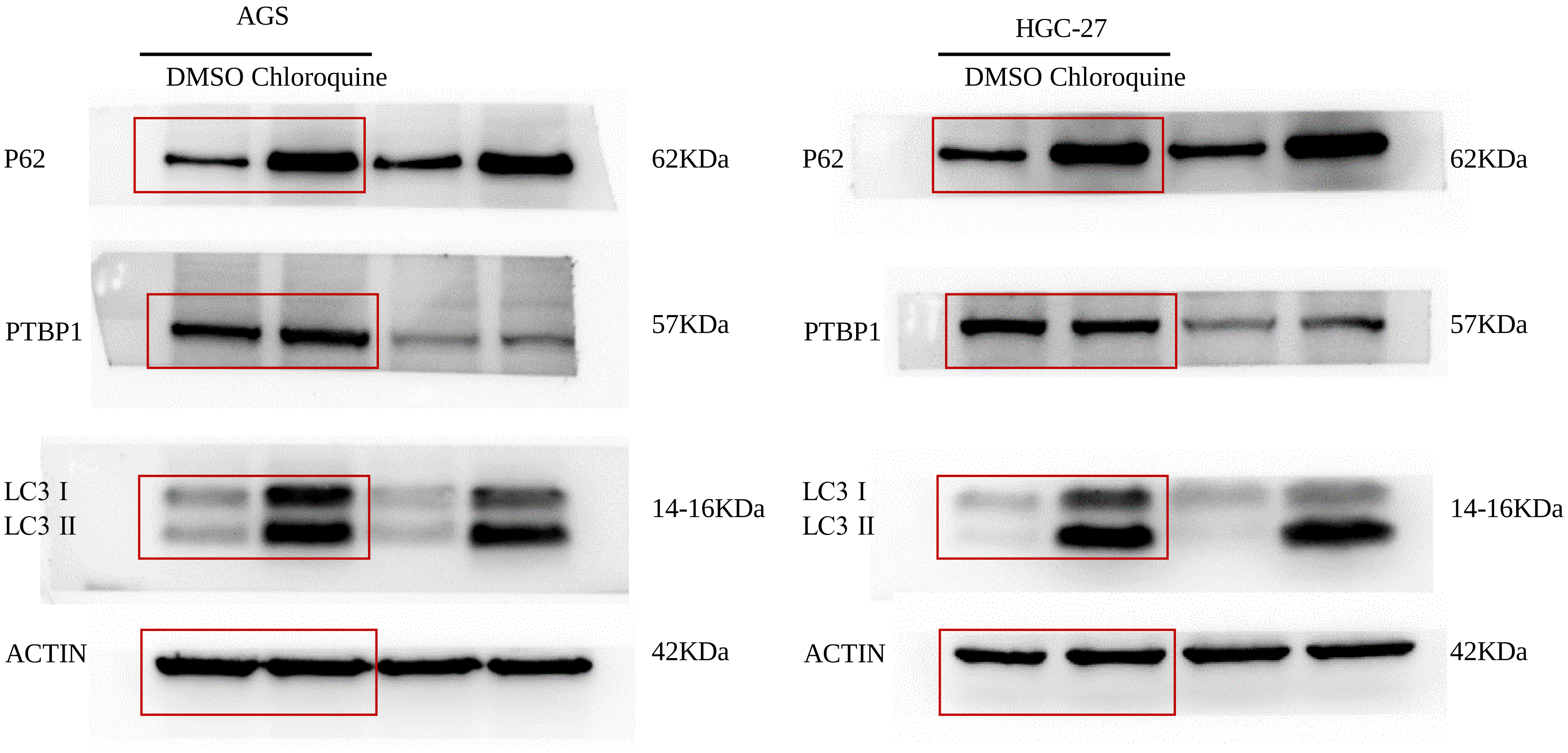

Supplement: Supplementary file 3 — Supplementary material 3: File S3. Uncropped blots for figures. [file 11658_2024_626_MOESM3_ESM.pdf]
